# A paradoxical population structure of *var* DBLα types in Africa

**DOI:** 10.1371/journal.ppat.1012813

**Published:** 2025-02-04

**Authors:** Mun Hua Tan, Kathryn E. Tiedje, Qian Feng, Qi Zhan, Mercedes Pascual, Heejung Shim, Yao-ban Chan, Karen P. Day

**Affiliations:** 1 Department of Microbiology and Immunology, Bio21 Institute and The Peter Doherty Institute for Infection and Immunity, The University of Melbourne, Melbourne, Australia; 2 School of Mathematics and Statistics / Melbourne Integrative Genomics, The University of Melbourne, Melbourne, Australia; 3 Committee on Genetics, Genomics and Systems Biology, The University of Chicago, Chicago, Illinois, United States of America; 4 Department of Biology, New York University, New York, New York, United States of America; Nanyang Technological University, SINGAPORE

## Abstract

The *var* multigene family encodes *Plasmodium falciparum* erythrocyte membrane protein 1 (PfEMP1), central to host-parasite interactions. Genome structure studies have identified three major groups of *var* genes by specific upstream sequences (upsA, B, or C). *Var* with these ups groups have different chromosomal locations, transcriptional directions, and associations with disease severity. Here we explore temporal and spatial diversity of a region of *var* genes encoding the DBLα domain of PfEMP1 in Africa. By applying a novel ups classification algorithm (*cUps*) to publicly-available DBLα sequence datasets, we categorised DBLα according to association with the three ups groups, thereby avoiding the need to sequence complete genes. Data from deep sequencing of DBLα types in a local population in northern Ghana surveyed seven times from 2012 to 2017 found variants with rare-to-moderate-to-extreme frequencies, and the common variants were temporally stable in this local endemic area. Furthermore, we observed that every isolate repertoire, whether mono- or multiclonal, comprised DBLα types occurring with these frequency ranges implying a common genome structure. When comparing African countries of Ghana, Gabon, Malawi, and Uganda, we report that some DBLα types were consistently found at high frequencies in multiple African countries while others were common only at the country level. The implication of these local and pan-Africa population patterns is discussed in terms of advantage to the parasite with regards to within-host adaptation and resilience to malaria control.

## Author summary

The World Health Organisation reported 233 million clinical cases in the African region in 2022, accounting for 94% of global malaria cases. The *var* multigene family encodes an important virulence factor of the dominant malaria parasite, *Plasmodium falciparum*, and is often reported with extreme genetic diversity, particularly in areas with high malaria transmission. Here, we report on the diversity and prevalence of a *var* fragment known as DBLα types in several populations in Africa, using a novel algorithm developed to classify DBLα types into three major ups groups (A, B, C). We found that there were individual DBLα types that persist in a local area through time, occurring at stable high, moderate, or low frequencies in the population. We showed that this frequency structure was possible because every infection also exhibited balanced structures, suggesting that there is pressure for a parasite to maintain common and rare types in its genome. We also identified “local” DBLα types that were present predominantly in a single location but were absent/rare in other locations. By uncovering stable patterns within this complex system, our study provides new insights into the genetics of these important genes to bridge our understanding of interactions of parasites with human hosts.

## 1. Introduction

Antigenic variation is an immune evasion strategy that has evolved in viral, bacterial, fungal, and protist pathogens. In many taxa, it is mediated by differential expression of hyperdiverse variant antigen genes to rapidly change surface antigens. These antigenic switches facilitate within- and between-host transmission.

*Plasmodium* parasites of the subgenus Laverania infecting gorillas, chimps, and humans have a multigene family designated *var* [1,2]. In *Plasmodium falciparum*, the most virulent species infecting humans, the *var* multigene family encodes the major variant surface antigen of the blood stages, designated PfEMP1 [3]. This molecule undergoes clonal antigenic variation such that a single infected red blood cell expresses only one of the 40 to 60 *var* genes in a parasite’s genome at the trophozoite stage [4,5]. PfEMP1 facilitates sequestration by cytoadhesion to several host receptors to avoid splenic mechanisms of parasite clearance [6–8]. Consequently, certain variants and *var* gene motifs have been identified as virulence factors [9–16].

*Var* genes exist in multiple architectural types that encode the specific structural arrangements of PfEMP1 domains [17]. These genes evolve by proposed mechanisms involving homologous recombination where sequences encoding specific domains recombine with a homologous domain rather than heterologous [18,19], maintaining a cassette-based structure [20]. Despite the importance of *var* genes to the biology of *P*. *falciparum*, there have been limited studies of the population genetics of these genes. Currently, they are excluded from genome projects due to challenges in the assembly of this hyperdiverse multigene family. To date there has been only one large-scale *var* gene assembly project publicly available [21] describing aspects of the population genetics of *var* gene domains at the continent level, showing global population structure.

**Fig 1:**
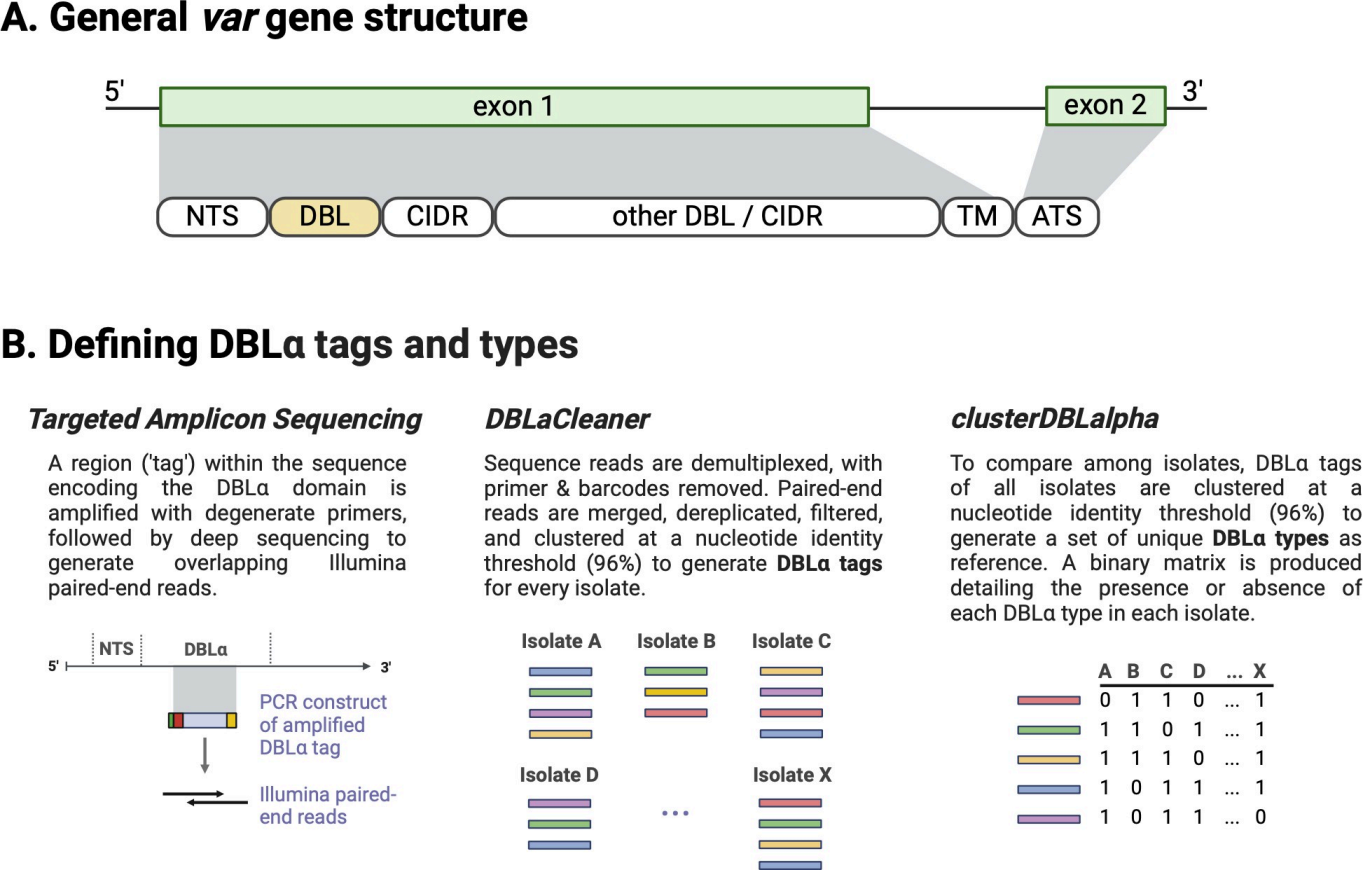
*Var* genes and workflow to generate *var* DBLα sequences. **(A)** The general *var* gene structure consists of two exons, which encode the extracellular and intracellular portions of the PfEMP1 protein. The first exon typically consists of an N-terminal segment (NTS) on the 5’ end, followed by sequences encoding multiple semi-conserved domains such as Duffy binding-like (DBL) and cysteine-rich interdomain region (CIDR) domains that, when combined, make up various *var* domain compositions and structures. Based on the upstream promoter sequence, *var* genes in this multigene family can be further divided into four subgroups of upsA, upsB, upsC, and upsE. **(B)** Defining the DBLα tag region and a description of the analysis workflow to generate DBLα types.

To facilitate data collection on the diversity of these genes in local endemic areas, where tens of thousands of variants have been shown to exist in relatively limited sampling of infected individuals, a strategy has emerged to circumvent the need for complete *var* gene sequences by exploiting the cassette-like structure of these genes [22]. This approach focuses on the Duffy-binding-like alpha (DBLα) domain that is encoded by all *var* genes (Fig 1A), with the exception of one specific *var* gene involved in pregnancy-associated malaria [20]. The DBLα domain is one of the most diverse [19] and has been shown to be immunogenic [14,23]. This domain also potentially plays a role in adhesion, either in itself or in linkage with a proximal domain. Specifically, except for the isolate-transcendent *var* genes (i.e., *var1*, *var2csa*, *var3*), the extracellular N-terminal PfEMP1 head structure consists of a DBLα domain and a cysteine-rich interdomain region (CIDR) (i.e., a DBLα-CIDR tandem) [17,20] and can influence ligand binding and disease pathogenicity [24,25]. Analysis of the relationship between DBLα types and *var* exon 1 sequences has shown that majority of DBLα types (especially in the non-upsA group, >75%) represent a unique *var* gene each in areas with high malaria transmission [26], which serves as the basis for a laboratory protocol for targeted amplicon sequencing of “DBLα tags” (i.e., *var*coding [22]), ultimately generating reference “DBLα types” [22,27] (Fig 1B).

Global studies have shown high diversity and geographic variation in DBLα types [21,28] but with a minority of DBLα types or *var* genes found to be conserved globally at high frequencies (e.g., the top 100 frequent DBLα types in 1,248 isolates across Africa, Asia/Oceania, and South America [28]; conserved *var* gene in 36 of 714 African and Asian parasites [29]). Here, we describe the population frequencies of individual DBLα types in African *P*. *falciparum* populations in high transmission, where most of the global malaria burden is concentrated. We focused on sampling from the African continent where we have the oldest parasite populations with the greatest diversity of DBLα types [21,28] and extensive geographic variation in the human genome, which may result in selection of local patterns of parasite diversity. Analyses in high-transmission African settings, where we have observed a parasite population structure of largely non-overlapping DBLα repertoires [22,30], also avoids confusing stable frequencies of individual DBLα types that result from clonality or high relatedness as selection.

Genome structure studies of the *var* multigene family have shown that *var* genes can be divided into groups of A, B, C, and E, with a minority of genes grouped into two intermediate groups of B/A or B/C, based on upstream promoter sequences [31]. The three major ‘ups’ groups of upsA, upsB, and upsC are associated with different chromosomal locations, transcriptional directions, and sequences [1,4,31,32]. Associations of expression of specific ups groups and disease outcomes have also been found, e.g. upregulated expressions of upsA *var* genes have been shown in cerebral and severe malaria patients, compared to uncomplicated malaria [33–36].

Given these differences, we sought to explore the population genetics of DBLα types by analysis of spatial and temporally collected datasets stratified by ups groups. This was done using a novel ups classification algorithm (*cUps*) introduced in this paper, capable of classifying DBLα types further into upsA, upsB, and upsC groups. Using these short sequences avoids the challenge and expense of long-range sequencing of hyperdiverse *var* genes in multiclonal infections. We analysed the temporal patterns of 62,158 DBLα types and their associated frequencies from seven serial cross-sectional surveys of a local parasite population in Ghana over six years. Within this Ghanaian population, we identified a paradoxical population structure of DBLα types where types in all three major ups groups were maintained at different levels of “common-ness” at low, moderate, high, and extreme frequencies in the population, and this pattern also persisted through time. This observation was possible because every isolate and parasite repertoire also comprised DBLα types occurring at low-to-extreme population frequencies. Based on a further frequency analysis of 79,192 DBLα types from locations in Ghana, Gabon, Malawi, and Uganda, we noted that there were DBLα types that were common in a specific country but not necessarily as common across wider geographical scales, which can suggest local adaptation of the parasite to geographically-diverse receptors and immune response genes of humans in Africa.

## 2. Results

### 2.1 Description of time-series cross-sectional surveys in Bongo, Ghana

This study analysed publicly-available DBLα tag sequence data from an interrupted time-series study design (i.e., referred to as the “Malaria Reservoir Study” (MRS)) (Fig A in S1 Text) [22,37–41]. This MRS dataset consists of sampling at seven time points from 2012 to 2017 at the end of wet (October) or dry (May/June) seasons. Each time point represented an age-stratified cross-sectional survey of approximately 2,000 participants per survey (ages from 1 to 97 years old) from two proximal catchment areas (Vea/Gowrie and Soe, with a sampling area ~60 km^2^) in Bongo District, located in northern Ghana. Surveyed participants (i.e., isolates) represented repeat sampling of ~15% of the total population that reside in the two catchment areas in Bongo District at a time (Table A in S1 Text). This area is characterised by high, seasonal malaria transmission and has undergone several types of malaria control interventions, including long-lasting insecticidal nets (LLINs) and indoor residual spraying (IRS) that reduced transmission [22,40], as well as seasonal malaria chemoprevention (SMC) that reduced the burden of infection in children younger than 5 years old [22]. A total of 62,158 representative DBLα types were found in 3,166 asymptomatic isolates from seven surveys (S1 to S7) and served as the dataset for exploring DBLα type frequencies in the parasite population in Bongo (Table A in S1 Text). The number of DBLα types per isolate was not impacted by sequencing depth (Fig B in S1 Text).

To clearly define terminologies, in a high-transmission setting, the asymptomatic “parasite population” typically consists of “isolates” infected by one or more “unique parasite genomes”. This complexity of infections is indicated by “multiplicity of infection” (MOI), where an isolate with MOI = 1 would represent a single unique parasite genome. Hence, at MOI = 1, an isolate’s DBLα repertoire is synonymous to a parasite’s DBLα repertoire. Conversely, at MOI > 1, an isolate’s DBLα repertoire would encompass > 1 parasites’ DBLα repertoire.

### 2.2 A novel ups classification algorithm based on DBLα sequences

**Fig 2:**
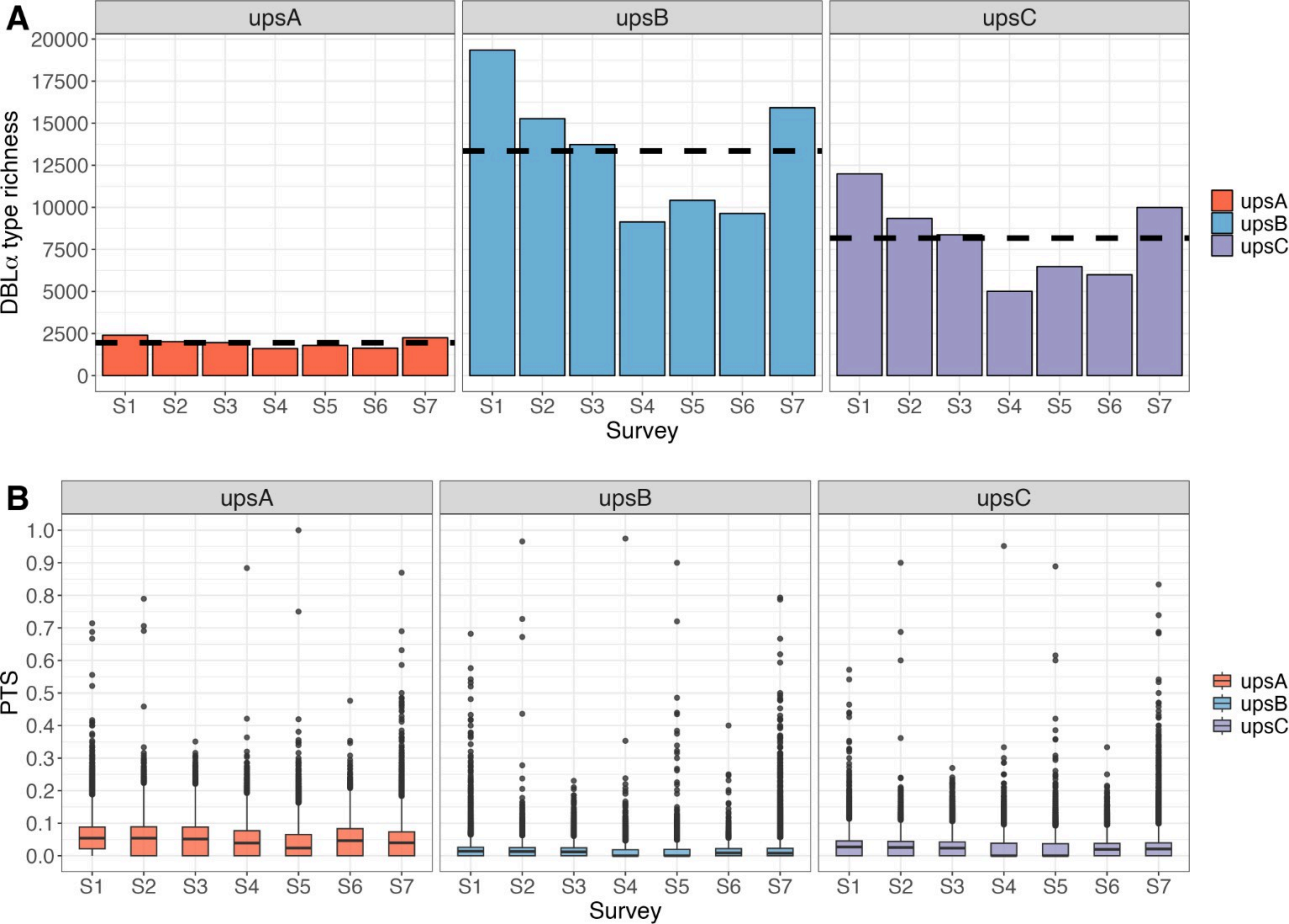
Classification of DBLα types into ups groups (upsA, upsB, upsC) for each of the seven MRS surveys in Bongo, Ghana [Malaria Reservoir Study (MRS)]. Higher DBLα type richness (i.e., number of unique DBLα types) and lower repertoire overlap is observed in upsB and upsC groups, relative to upsA. **(A)** DBLα type richness, where the horizontal dashed lines show mean richness per ups group. **(B)** Genetic similarity / overlap of isolate repertoires by pairwise type sharing (PTS). Box plots indicate the median value (centre line), interquartile ranges (IQR, upper and lower quartiles), 1.5× IQR (whiskers), and outliers (points).

There are limitations to current approaches to classify DBLα types into their respective ups groups. Ups grouping of *var* genes with phylogeny-based methods typically require 5’ UTR sequences that are not generated from targeted amplification [20]. Without access to assembled genomes, as is common in many large-scale targeted amplicon sequencing projects, a current approach exists to classify DBLα types into ups groups, but is limited to only differentiating upsA from non-upsA types by the DBLα domains identified [42]. This study introduces ***cUps***, a novel algorithm for classifying DBLα types further into the different groups of upsA, upsB, and upsC (S2 Text), reporting higher levels of richness of upsB and upsC types, relative to upsA at the population level (Fig 2A). At the isolate level, the average isolate repertoire consisted of 20.9%, 48.6%, and 30.5% of upsA, upsB, and upsC DBLα types, respectively (Fig C in S1 Text). These proportions differ from those reported in [20] that estimated higher proportions of upsB and lower proportions of upsC in isolate repertoires, based on the average of seven genomes. The *cUps* algorithm showed a tendency to classify more upsB types as upsC types, and this is in line with validation results on the algorithm’s specificity and sensitivity, elaborated in S2 Text. A reduced analysis that involved thresholding for DBLα types with higher confidence in classification yielded similar patterns of observation we report in this manuscript. Genetic similarity of isolate repertoires by pairwise type sharing (PTS) remained low for all ups groups (median PTS: 0.0455 (upsA), 0.0094 (upsB), and 0.0215 (upsC)) (Fig 2B), where PTS values from 0 to 1 represent the range from unrelated to identical isolate repertoires. The 62,158 representative DBLα types from the seven combined MRS surveys were classified into upsA, upsB, and upsC groups (Table A in S1 Text). The differences in proportions of ups groups at the isolate *vs* population levels are attributed to the negative relationship between PTS and richness, as a higher level of upsA DBLα type sharing among isolates will result in a lower proportion of unique representative upsA DBLα types in the population.

### 2.3 Stability of DBLα types and frequencies in the local Bongo population

The frequency of a DBLα type is defined as the proportion of isolates with this DBLα type and was calculated in the context of individual surveys (i.e., “survey-specific frequency”) or of the average of seven surveys (i.e., “survey-averaged frequency”). These frequencies were further categorised into four classes to indicate different levels of “common-ness” with ranges given in interval notations: ***low*** (0%, 1%), ***moderate*** [1%, 5%), ***high*** [5%, 10%), and ***extreme*** [10%, 100%] frequencies. While it is well reported that DBLα types in the upsA group are generally more commonly shared relative to DBLα types in the upsB and upsC groups, this study identified individual DBLα types that were stable in the context of sequences and frequencies in all three ups groups at the population level, as described in the following subsections.

#### 2.3.1. A subset of DBLα types was present in many isolates at each surveyed time point

**Fig 3:**
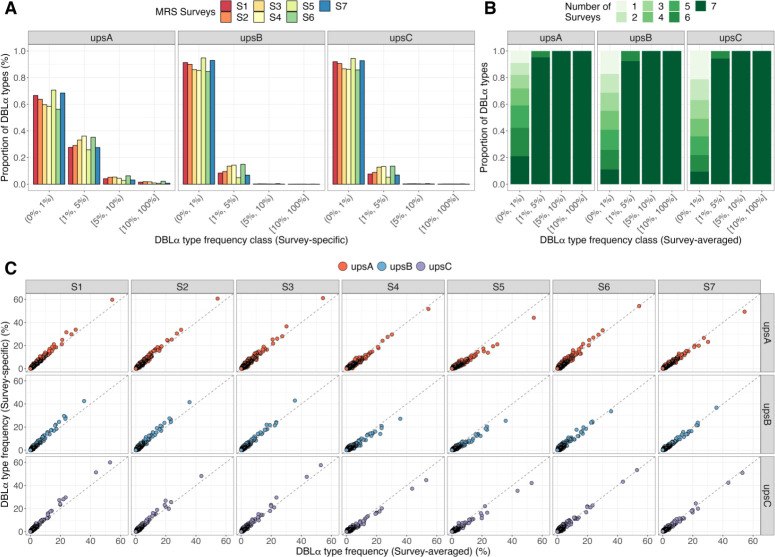
Stable DBLα type sequences and frequencies are observed in a local population and through time [Malaria Reservoir Study (MRS)]. **(A)** Binned into categorical frequency classes with ranges given in interval notations of low (0%, 1%), moderate [1%, 5%), high [5%, 10%), and extreme [10%, 100%], distributions of survey-specific DBLα type frequencies show that most DBLα types are present at low frequencies, followed by those at moderate frequencies, and a minority of types present at high or extreme frequencies. These proportions are listed in Table B in S1 Text. The most-frequent DBLα types in the upsA, upsB, and upsC groups were detected at survey-specific frequencies of 61.1%, 42.9%, and 62.0%, respectively. **(B)** Based on the number of surveys DBLα types are observed in, DBLα types that are present at moderate-to-extreme survey-averaged frequencies (≥1%) are shown to also persist through time. This was observed for all three ups groups. This is also true for DBLα types present at moderate-to-extreme survey-specific frequencies (Fig C in S1 Text). **(C)** Strong, positive correlation is shown between survey-specific frequencies and survey-averaged frequencies of individual DBLα types (points), coloured by ups groups, indicative of stable DBLα type frequencies through time.

Most DBLα types occurred at low frequencies, found in <1% of isolates. For all three ups groups, the second largest subsets of DBLα types were found to be moderately common, present at 1% to 5% frequencies in each survey, followed by smaller subsets of highly or extremely common DBLα types, exceeding 5% or 10% frequencies, respectively (Fig 3A). The most common DBLα types in the upsA, upsB, and upsC groups were detected at survey-specific frequencies of 61.1%, 42.9%, and 62.0%, respectively. In the different surveys, this study identified hundreds to thousands of DBLα types with moderate-to-extreme frequencies in all three ups groups (upsA: 526 to 801 types per survey, upsB: 540 to 1,918 types per survey, upsC: 365 to 1,121 types per survey). This translates into different proportions of DBLα types in each ups group, owing to the higher DBLα type richness of upsB and upsC groups (upsA: 29.3% to 43.7% per survey, upsB: 5.2% to 15.5% per survey, upsC: 5.6% to 14.3% per survey) (Table B in S1 Text).

#### 2.3.2. Moderate-to-extremely common DBLα type sequences persisted in the population through time

*Var* genes were thought to be inherently unstable, based on *in vitro* evolution experiments, with the DBLα domain reported with the highest rate of recombination [19]. Contrary to this report, this study observed that specific DBLα types with moderate-to-extreme frequencies in each survey were seen persisting through time for all ups groups (Fig 3B, Fig D in S1 Text). All highly or extremely common DBLα types in a survey were also present across all seven surveys. The majority of those moderately common DBLα types were found in all seven surveys, with a smaller subset found in four to six surveys. Notably, DBLα types found in six surveys were missing mostly in the post-IRS intervention survey (S5). On the other hand, the remainder of DBLα types present at low frequencies were found in the range of one to seven surveys.

#### 2.3.3. DBLα type frequencies were stable through time

The population frequencies of DBLα types were also maintained across multiple surveys. In all ups groups, a strong positive correlation between survey-specific frequencies and survey-averaged frequencies of DBLα types is shown, indicating that DBLα types present at high frequencies in individual surveys were also present at high frequencies consistently through time (Fig 3C, Fig E in S1 Text). Some statistically significant variation across surveys of the frequencies of the 500 most common types was observed; however, this variation appears to come exclusively from surveys S4 and S5, which were affected by the IRS intervention (Fig F in S1 Text). Likewise, most moderately common DBLα types maintained stable frequencies across multiple surveys; no significant variation could be detected even in surveys S4 and S5. For these surveys affected by the IRS intervention, while rare types were lost and other types shifted to lower frequency classes, the rank of DBLα types by frequency did not change.

### 2.4 Isolate and parasite genome repertoires consisted of a mix of common and rare DBLα types

**Fig 4:**
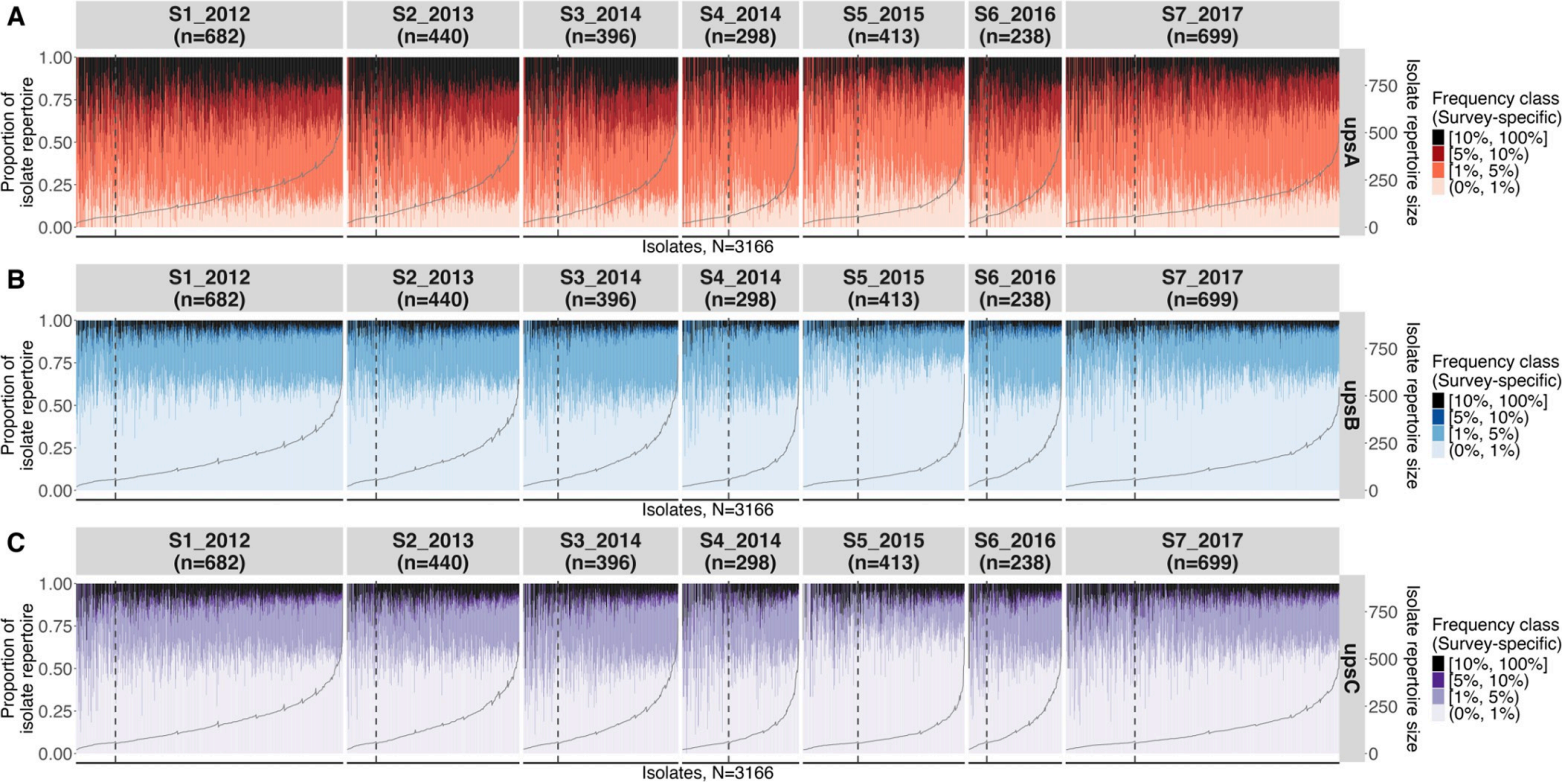
A consistent pattern in per-isolate frequency profiles for upsA, upsB, and upsC DBLα types reveals a new aspect of genome structure [Malaria Reservoir Study (MRS)]. For all ups groups, the per-isolate frequency profiles comprised of a mix of low-to-extreme survey-specific frequency classes and are consistent across isolates within the same survey, regardless of isolates’ infection complexities. Per-isolate frequency profiles are shown here by ups group **(A)** upsA, **(B)** upsB, and **(C)** upsC and by survey (vertical panels), where the ‘n’ value in the label represents the number of isolates per survey. Vertical bars represent individual isolates, ordered in increasing MOI and isolate repertoire size (grey line, secondary y-axis). Colours indicate survey-specific frequency classes with ranges given in interval notations of low (0%, 1%), moderate [1%, 5%), high [5%, 10%), and extreme [10%, 100%], and the proportions of these frequency classes within each isolate is shown on the primary y-axis. Within each panel, dashed vertical lines separate monoclonal (MOI = 1, left of line) and multiclonal (MOI > 1, right of line) isolates, whereby the monoclonal infections reflect the composition within actual parasite repertoires.

While isolate DBLα repertoires in high-transmission populations have been reported to be unrelated and largely non-overlapping [22,30,41–43], there has not been a detailed exploration of the composition of DBLα types within an isolate and their associated frequencies in the population (i.e., ‘per-isolate frequency profiles’). These per-isolate frequency profiles comprised of a mix of all frequency classes, based on survey-specific frequencies, and were consistent across isolates within the same survey, regardless of isolates’ infection complexities (Fig 4, Fig G in S1 Text). Fitting a binomial regression to per-isolate proportions of DBLα types in each frequency class did not detect any underdispersion, which would arise from a force of balancing selection to maintain these proportions at a fixed level in each isolate. Importantly, observing these per-isolate frequency profiles for monoclonal isolates (i.e., MOI = 1) indicate that these per-isolate frequency profiles reflect the repertoire composition within actual parasite genomes (Fig 4). We also confirmed this observation of per-isolate frequency profiles using an independent DBLα sequence dataset [21,26,44], extracted from *var* genes of isolates sampled from Navrongo in Ghana, situated ~30 km adjacent to Bongo District (Fig H in S1 Text). The use of data obtained from two different methods (i.e., targeted amplicon sequencing and whole genome sequencing) yielded similar observations, providing confidence that these patterns were not introduced from the PCR protocol used in *var*coding.

Isolates’ upsA frequency profiles consisted of mostly moderate-to-extremely common DBLα types and relatively small proportions of rare or unique DBLα types. In contrast, isolates’ upsB and upsC frequency profiles consisted of large proportions of DBLα types in the low frequency class, followed by those in the moderate frequency class. The introduction of malaria control interventions did not perturb these frequency profiles, which maintained the composition of different frequency classes but in different proportions. In surveys of the population affected by interventions (e.g., S4 and S5, during and post-IRS), per-isolate frequency profiles generally trended toward a larger proportion of relatively rare DBLα types and smaller proportions of relatively common types within each isolate (Fig 4). However, as noted above, the rank of DBLα types by population frequencies were maintained.

To investigate the extent of sharing of DBLα types within each of the four frequency classes, genetic similarity among pairwise isolate repertoires was calculated (i.e., pairwise type sharing (PTS) values). As expected, PTS values increased as rare DBLα types were excluded (Fig I in S1 Text). Interestingly, even when considering only extremely common DBLα types, median PTS values remained generally low (median PTS of 0.02, 0.05, 0.13, and 0.20 when considering DBLα types at >0%, ≥1%, ≥5%, ≥10% survey-averaged frequencies, respectively, across all surveys). This indicates that, even though every isolate repertoire contained a proportion of types that were present in many isolates in the population, identical sets of common DBLα types were rarely observed. When evaluating DBLα types exclusively in the different ups groups, shifts in PTS distributions were more substantial for the upsA or upsC groups relative to the upsB group, consistent with the lower DBLα richness in the two former groups.

### 2.5 Local DBLα types were detected in individual African countries

**Fig 5:**
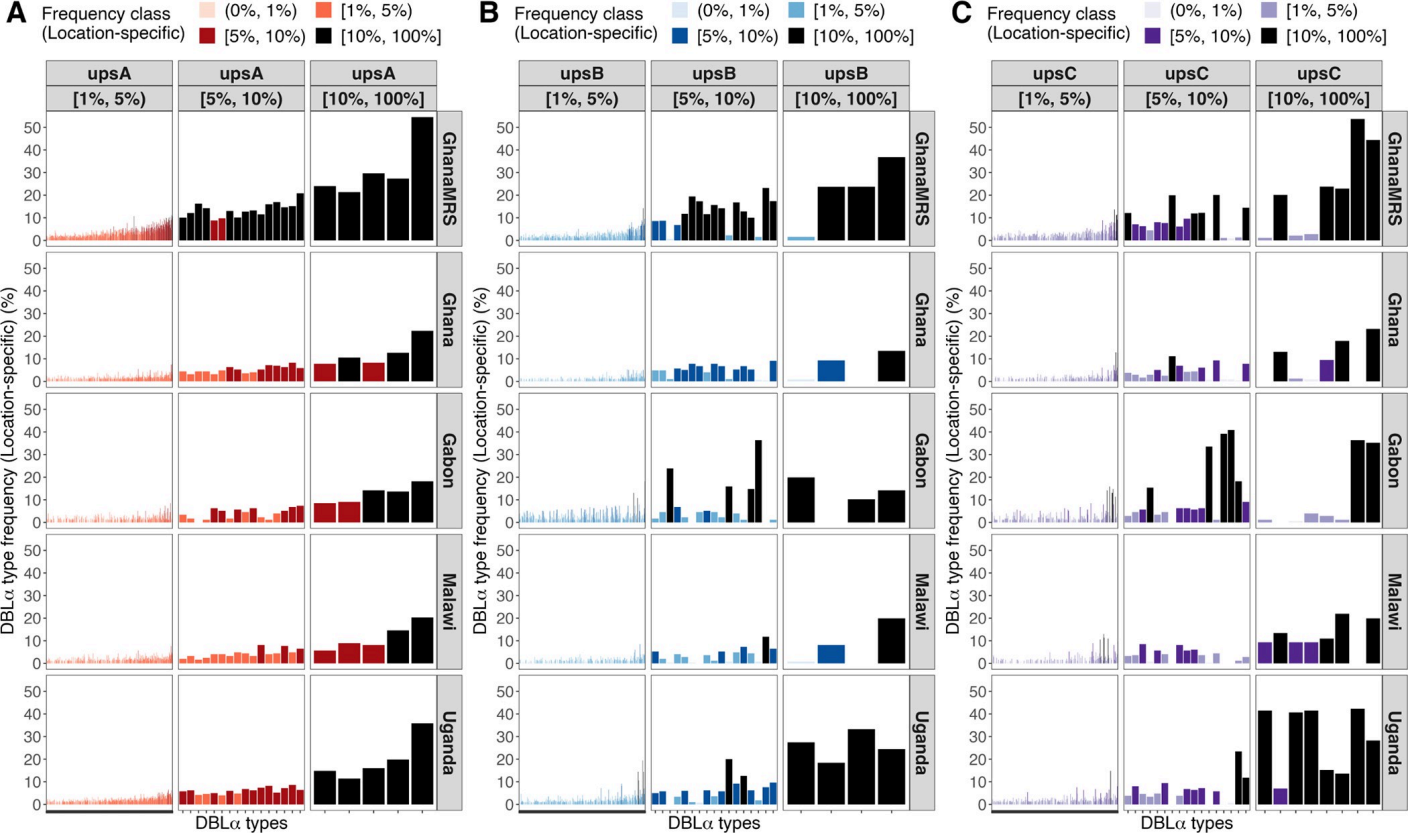
DBLα type frequencies within Ghana, Gabon, Malawi, and Uganda reveal location-specific signatures [geographical analysis]. These location-specific signatures are more apparent in the upsB and upsC groups, with DBLα types that are present predominantly in a single location but are absent or at low frequencies in other locations. Shown here for **(A)** upsA, **(B)** upsB, and **(C)** upsC groups are DBLα types with moderate-to-extreme location-averaged frequencies (≥1%), ordered in increasing location-averaged frequencies. Vertical panels represent location-averaged frequency classes and horizontal panels represent locations in Africa. Bars indicate the location-specific frequencies of individual DBLα types, further coloured according to location-specific frequency classes with ranges given in interval notations of low (0%, 1%), moderate [1%, 5%), high [5%, 10%), and extreme [10%, 100%].

A separate geographical study of DBLα types and frequencies in multiple African countries (i.e., “locations”) representing West Africa (Ghana, Gabon), Central Africa (Malawi) and East Africa (Uganda) was conducted based on 79,192 DBLα types found in 4,561 isolates (Table A and Fig A in S3 Text) [21,26,44]. Similarly, this geographical study showed that the majority of DBLα types in each location were present at low frequencies with smaller proportions seen at relatively higher frequencies. While most of the more common upsA DBLα types were present at relatively stable frequencies in most of the analysed countries, DBLα types in the upsB and upsC groups were common at either the continent or local levels (Fig 5). This was evident from finding common upsB and upsC DBLα types that were present predominantly in a single location but were absent or uncommon in other locations, suggesting local selection (Fig 5, Fig B in S3 Text). As observed for isolates in the Ghana MRS study, per-isolate frequency profiles in these different locations also consisted of a mix of common and rare DBLα types (Fig C in S3 Text).

It is worth remembering that these common DBLα types made up the minority of all DBLα types in every ups group, especially in the upsB and upsC groups that formed the majority of DBLα types in a population as well as in an isolate’s repertoire. An exploration of the relationship between DBLα types and *var* exon 1 sequences revealed that DBLα types at high or extreme frequencies tended to be associated with multiple different *var* exon 1 sequences (Fig D in S3 Text), indicating that other parts of the gene were still diversifying even though the DBLα types were maintained in the population. For some of these DBLα types with 1-to-many DBLα-*var* relationships, analysis of sequence similarity between *var* exon 1 sequences that shared the same DBLα type suggested that the majority of these *var* exon 1 sequences exhibited low shared identity and therefore appeared to represent actual different genes (Fig D in S3 Text), with a smaller subset potentially being alleles of a same gene [26]. In a highly-dynamic system where sequences encoding the DBLα domain have been shown *in vitro* to exhibit the highest recombination rate [19], the maintenance of specific DBLα types at high frequencies and through extensive durations is characteristic of balancing selection. We make clear that this study describes the conservation of DBLα types, where we envisage evolution of domains or cassettes, but not necessarily the conservation of *var* genes.

### 2.6 Factors maintaining DBLα type sequences and frequencies remain unknown

The geographical study considered a few possible factors to explain these common DBLα types, focusing specifically on 50 and 17 DBLα types in the high and extreme location-averaged frequency classes, respectively (Fig E in S3 Text). While positional information is unavailable for the DBLα types analysed in this study, sequence alignments showed that only six of the 67 DBLα types are homologous to DBLα tags of *var* genes on specific *P*. *falciparum* chromosomes 4, 6, 7, and 8 that were previously detected at high frequencies in various populations, potentially attributed to selective sweep events associated with antimalarial drug resistance [21]. Furthermore, some of these DBLα types with high or extreme frequencies were identified as homologs to the DBLα tags of five *var* genes in *P*. *praefalciparum*, the closest living sister species of *P*. *falciparum* that naturally infects gorillas [2, 45]. Homologs to the DBLα tags of two *var* genes of another ancestral Laverania species, *P*. *reichenowi*, were identified but were present at low-to-moderate frequencies in this geographical dataset (frequencies range from 0.57% to 2.40%). No homologs to the DBLα tags of *P*. *gaboni var* genes were identified. While some of these factors can explain the reason a few of these sequences were conserved, the majority of these DBLα types with relatively high frequencies were still unaccounted for.

With respect to published studies on globally-conserved DBLα types or *var* genes, homologs to 84 of the globally-conserved DBLα types [28] were identified in this study, with 37 of these DBLα types found in the two highest frequency classes. Furthermore, in the context of general prevalence in the analysed African locations, 30 of these 37 DBLα types were found in all four locations, six in three locations, and only one was found in a single location. The homolog to the DBLα tag of a conserved *var* gene that was reported in a Gabonese parasite isolate [29] was present at high-to-extreme frequencies (ranging from 7.0% to 20.1% in different locations) and present in all locations except, strangely, in Gabon itself. Additionally, the homolog to the DBLα tag of a *var* gene that was reported to be expressed in sporozoites and potentially play a role in hepatocyte infection [46] was present at only <1% frequency in three locations. These annotations are available in the data tables online (see Data Availability section).

## 3. Discussion

Extensive DBLα type diversity is reported in areas with high malaria transmission, generated by meiotic and mitotic recombination [18,19,47–49] with parasite repertoire diversity driven by frequent outcrossing in the mosquito vector [50,51], such that we would not expect conservation of types. Here we report a paradoxical population structure of DBLα types, where types are seen stable through time and can be found in a population at various frequencies, be it low, moderate, or high, in all three major ups groups. Underlying and maintaining this observed population structure of DBLα types in a local endemic area is the frequency profile of DBLα types within every isolate repertoire, whether mono- or multiclonal. This implies a common genome structure. Whilst multiclonality could potentially lead to the underestimation of sharedness and conversely the overestimation of uniqueness, we perceive this to be of minimal effect due to the reported lack of DBLα repertoire overlap amongst likely monoclonal isolates [30] and the large effective population size in high transmission [22,41]. The consistency of these per-isolate frequency profiles, seen within our Ghana MRS population and the independent African DBLα datasets [21], therefore suggests that each isolate repertoire must have a combination of common and rare types for the three major ups groups while still maintaining limited overlaps with other isolates in the population overall. It is obvious from prior work that rare types would be non-overlapping [22,30], but what is striking in this analysis is that the common types of all three major ups groups were not co-occurring in the same isolates; low PTS was still observed indicating the lack of sharing of common types (Fig I in S1 Text). We hypothesise that temporal stability of DBLα types and frequency structures in the population may be maintained by human host factors involved in host-parasite interactions.

We propose that DBLα types that exist in low-to-moderate frequencies in the parasite population serve to provide the parasite with options to benefit within-host survival by antibody-mediated immune evasion while the minority of DBLα types occurring at higher frequencies in the parasite population may reflect adaptations to maintaining infection in a local human host population. We would interpret this observed structure to be a consequence of different rates of recombination of DBLα types resulting in these common *vs* rare types. Alternatively, there are multiple selective forces to maintain DBLα types at such varying frequencies in a parasite population. These would include a variety of host adhesion receptors and immune response genes. Our finding of local signatures of conservation in multiple African countries supports the possibility of local adaptation of individual DBLα types to the human host population, given that there are reports of geographic variation in host adhesion and immune receptors (e.g., [52–54]) and examples of co-evolution [55,56].

Stochastic simulations and network analyses have provided clear evidence for a role of immune selection or negative frequency-dependent selection resulting from specific immune memory, which is a form of balancing selection, in shaping antigenic diversity within natural populations [37]. As antibody-mediated immunity plays a significant role in recognition of PfEMP1 variants, we hypothesise that another possible driver of balancing selection is the arm’s race between the parasite PfEMP1 variants and host HLA class II haplotypes [55,57–59]. Similar to our finding of local signatures of DBLα type conservation against a highly-diverse background, there are also geographic differences in HLA class II alleles across the African continent, with allele frequencies also ranging from low to high [60–62]. Immune evasion related to local HLA class II alleles would select for varying frequencies of DBLα types. The paradoxical population structure of DBLα may also be shaped by underlying differences in host receptors of varying spatial niches and if not, this domain could be in linkage disequilibrium with other proximal domains (e.g., CIDR) or genes vital to these roles. Future studies of co-evolution must take into consideration that domains or domain cassettes appear to evolve independently by recombination [20].

The observed parasite genome structure composed of DBLα types with varying frequency classes has significant implications for malaria surveillance and control. The removal of parasite genomes from a population through intervention does not lead to the loss of the same proportion of DBLα types, as the initial removal of parasite genomes involves the loss of rare DBLα types mostly whereas common DBLα types persist until a high proportion of genomes are lost (Fig E in S1 Text). Thus, with interventions, diversity or richness of DBLα types would be seen to decrease in a non-linear manner relative to the removal of parasite genomes from a population (Fig J in S1 Text). Breaking this pattern towards high relatedness or clonality is indicative of a system transitioning into a low-transmission setting and thus could be diagnostic for elimination efforts [63].

In conclusion, the paradoxical population structure of DBLα types created by these consistent per-isolate frequency profile patterns is striking and suggests that maintaining such frequency profiles within a parasite repertoire is advantageous to the parasite. Having a range of rare to common types within each major ups group may allow malaria parasites to adapt to host factors in order to persist through the dynamics and competition within and between hosts.

These observations encourage us to identify the role of host genetic factors in selecting these stable frequencies. Of further interest for investigation is the significance of DBLα frequency profiles within all ups groups in a parasite genome in understanding the hierarchy of *var* gene expression in the human host [64], as well as future studies on gene expression levels and immunity structured according to these specific common *vs* rarer DBLα types within each ups group.

## 4. Materials and methods

### 4.1 Data sources and types

Frequency analyses were performed based on a small ~450bp sequence region of a *var* gene that encodes a portion of the DBLα domain of PfEMP1 (i.e., DBLα tags) [65,66]. DBLα tag sequences included in this study were either generated from targeted amplicon sequencing or extracted from assembled *var* gene sequences. This made available DBLα tag datasets of varying sizes from Africa and Asia, which were clustered to generate representative DBLα types. However, the scope of this study on DBLα conservation was limited to African locations only, with higher transmission, because lower transmission areas may present a different context underlying conservation (e.g., clonality or smaller population sizes). Data in Africa were available from West Africa (Senegal, The Gambia, Guinea, Mali, Ghana, Gabon), Central Africa (DR Congo, Malawi) and East Africa (Uganda, Kenya) (Table A in S1 Text and Table A in S3 Text). However, most of these African countries were excluded due to limited dataset sizes (number of isolates < 100), resulting in a final analysis from four locations in Africa (i.e., Ghana, Gabon, Malawi, Uganda). Sources and methods that the different studies used to generate these DBLα tag datasets are described in the following subsections.

#### 4.1.1 DBLα tags from targeted amplicon sequencing data

Published DBLα tag datasets from three locations were generated from targeted amplicon sequencing (Table A in S1 Text and Table A in S3 Text). Amplicon sequencing of DBLα tag sequences typically involves PCR amplification of a small sequence region encoding the DBLα domain of PfEMP1 (Fig 1) using degenerate primers [65,66], followed by high-throughput sequencing on either the Illumina MiSeq platform (GhanaMRS) or on the 454 sequencing platform (Gabon, Uganda). These include sequences from:

- One area (Bongo) in Ghana: dataset spans seven time points (surveys) from 2012 to 2017 involving sampling of asymptomatic individuals at the end of multiple wet (October) and dry (May/June) seasons (GenBank BioProject accession number: PRJNA396962) [22,37–41].
- One area (Bakoumba) in Gabon: dataset included sampling of asymptomatic children in one year (GenBank accession numbers: KY328840–KY341897) [30].
- Six areas (Apac, Arua, Jinja, Kanungu, Kyenjojo, Tororo) in Uganda: dataset included sampling of clinical isolates over two years (GenBank BioProject accession number: PRJNA385208) [42].

#### 4.1.2 DBLα tags from assembled *var* gene sequences

Published *var* gene sequences (from isolates in Africa and Asia) were downloaded from the ‘Full Dataset’ published by [21]. DBLα tag sequences were identified and extracted from *var* gene sequences (regardless of *var* gene completeness) as described in [26]. Briefly, domain annotations provided by [21] were used to extract nucleotide sequences encoding the DBLα domain. These extracted sequences were further translated into the best reading frames and, using *hmmsearch* [67], the resulting amino acid sequences were further searched against positions 189 to 430 of the PFAM profile alignment (PF05424_seed.txt) to identify the ‘tag’ region (domain score cut-off of 60 and ≥100 aligned positions) and to ultimately extract the DBLα tag sequence that would have been amplified with degenerate primers [65,66]. Isolates were excluded if suspected as laboratory isolate (“Lab” or “Suspected_lab_strain”) or incorrectly-designated continent (“Continent_mismatch”) based on their metadata.

### 4.2 Clustering of DBLα tags into DBLα types

DBLα tags (Africa and Asia) were translated into amino acid sequences and any untranslatable sequences (i.e., stop codons in reading frame) were excluded. The remaining DBLα tags were combined and clustered with *clusterDBLa* v1.0 [37] using a 96% nucleotide identity threshold [68] to produce representative DBLα types. This also generated a binary matrix detailing the presence/absence matrix of every DBLα type in every isolate. Initially described in [68], the use of this threshold is further supported by the results shown in Fig 5 of Feng et al. [69] that identified the most frequent recombination breakpoint positions at approximately 0.25, 0.50, and 0.80 relative positions of DBLα types, suggesting that the risk of over-clustering of DBLα tags is higher only when we approach the ~80% threshold point. Previous work shown in Figure S3 (Data Sheet 1) of Tan et al. [26] further reported that varying this similarity threshold from 90% to 100% did not substantially affect the number of total DBLα types generated from isolates in Cambodia, Thailand, Ghana, and Malawi from the dataset of assembled *var* genes [21].

A separate analysis to test the impact of sequencing depth on the number of DBLα types was conducted. For isolates in the GhanaMRS dataset, median read support of DBLα tags was calculated for each isolate, revealing that higher sequencing depth did not lead to a greater number of DBLα types (Fig B in S1 Text).

### 4.3 Classification of DBLα types into domain classes and ups groups

The *classifyDBLα* v1.0 pipeline [42] was used to classify DBLα types into DBLα domain classes of DBLα0, DBLα1, or DBLα2, in order to confirm that sequences were indeed those encoding the DBLα domain of PfEMP1. In addition, a novel algorithm (***cUps****)* described in this study was used to classify DBLα types into the most probable ups group (i.e., upsA, upsB, or upsC), accompanied by assignment probability values. For each DBLα type, ups grouping was assigned according to the prediction with the highest assignment probability. We describe this novel classification algorithm below and in more detail in S2 Text. An implementation of the algorithm is available at https://github.com/qianfeng2/cUps.

Through the alignment and clustering of 2kb sequences upstream of *var* genes, followed by the classification *var* genes into ups groups by Neighbour-joining (NJ) and Markov clustering (MCL) methods (trees available in S2 Text), a reference dataset of DBLα tag sequences was generated from 846 *var* genes from 16 *P*. *falciparum* genomes (see S2 Text) [5,20]. We begin with this reference database of DBLα tag sequences with ups groups and DBLα domain subclasses known. For each category (ups group/DBLα subclass combination), we align the reference sequences in the category using Clustal Omega v1.2.4 [70], then fit a profile hidden Markov model [71] using HMMER v3.2.1 [67] with default settings.

For a given query sequence (representing a DBLα type), we calculate the likelihood of the query sequence being drawn from the profile HMM of each category, using the forward algorithm. The posterior probability for each category is then calculated using Bayes’ Theorem, with the prior probabilities of each category calculated from the reference database. Summing over DBLα domain subclasses gives the posterior probability for each ups group (i.e., assignment probability). The query sequence can be classified to the ups group with the highest assignment probability. A threshold may optionally be applied, so that sequences with highest assignment probability below the threshold categorised as `unclassified`. Alternatively, a summary statistic may weight each ups group by the assignment probability. This method is described in much more detail, with verification [72].

### 4.4 Exclusion of DBLα types, isolates, and populations from the final DBLα type dataset

Only the DBLα types that were successfully classified into a DBLα domain class (i.e., DBLα0, DBLα1, or DBLα2) were retained in the final dataset. Subsequently, isolates with < 20 DBLα types were also removed from dataset to ensure robust analyses downstream (Table A in S1 Text and Table A in S3 Text). Specifically for the time-series dataset from the GhanaMRS study in Bongo District, Ghana, submicroscopic or symptomatic isolates were additionally excluded from the dataset. Further, using *blastn* (≥96% nucleotide identity, ≥95% query coverage) [73], DBLα types with homology to isolate-transcendent *var1*, *var2csa*, and *var3* sequences (sequences from [20,21]) were excluded to remove putative DBLα types previously reported as isolate-transcendent [1,32]. Finally, given that frequency classes and profiles were calculated based on proportional frequencies, only locations with datasets of ≥ 100 isolates were retained. This resulted in the exclusion of six African countries from this study (“*” in Table A in S3 Text).

### 4.5 Estimation of multiplicity of infection (MOI)

For the GhanaMRS dataset, the number of sequenced non-upsA DBLα types per isolate (i.e., count of upsB and upsC) was converted to its estimated MOI using a published Bayesian approach (prior = “uniform”, aggregate = “pool”) [22]. This approach relies on the hyperdiversity of DBLα types, particularly those in the non-upsA groups [26], and the limited repertoire similarity [22].

### 4.6 Genetic similarity between pairwise isolate repertoires

The pairwise type sharing metric (PTS) [68] was used to estimate the overlap between pairwise isolate repertoires (e.g., isolates *i* and *j*). Specifically:

$$PTS=\frac{2*{shared}_{ij}}{{Size}_{i}+{Size}_{j}}$$

where *shared*_*ij*_ is the number of shared DBLα types between repertoires of isolates *i* and *j*, and *Size*_*i*_ and *Size*_*j*_ are the total number of DBLα types (i.e., repertoire sizes) of isolates *i* and *j*, respectively. A PTS value of 0 indicates the absence of sharing between two isolates whereas a PTS value of 1 indicates completely identical isolate repertoires.

### 4.7 Calculation of DBLα type frequencies and assignments into frequency classes

Depending on the analysis, a population can be the collection of isolates sampled at a specific survey or time point in the time-series analyses (i.e., by year or survey in the GhanaMRS dataset) or the collection of isolates sampled from a specific region or location/country in the geographical analyses. Raw frequencies of DBLα types were defined at the survey or location level in counts (i.e., number of isolates with a particular DBLα type in each survey or location). Raw frequencies were converted into proportional frequencies through division of count frequencies by the total number of isolates at a corresponding time point or location, leading to “survey-specific frequencies” or “location-specific frequencies”. Subsequently, these frequencies were further categorised into frequency classes with ranges given in interval notations: ***low*** (0%, 1%), ***moderate*** [1%, 5%), ***high*** [5%, 10%), and ***extreme*** [10%, 100%] frequencies.

Given the substantial differences in dataset sizes across surveys or locations (e.g., 499 isolates for Uganda *versus* 176 isolates for Gabon), simply summing isolates across datasets of multiple surveys or locations would bias total frequencies to reflect those of larger datasets. Hence, averaged frequencies were used instead as a means to normalise total frequencies by isolate counts in each survey or location (“survey-averaged frequencies” or “location-averaged frequencies”). For example, a DBLα type found in 10 out of 100 isolates for location A and 10 out of 500 isolates for location B would be reported to have 10% and 2% frequencies for locations A and B, respectively. A crude total frequency of 3.33% (20 of 600 isolates) would be more reflective of the frequency observed in location B even though the DBLα type was found at relatively high frequency at location A. In this instance, with normalisation, an averaged frequency of 6% would be estimated (12 of 200 isolates), reducing the bias towards larger dataset sizes. This normalisation method provides a less biased approach in identifying DBLα types that are found at high frequencies in one or more datasets but not necessarily uniformly across all datasets.

### 4.8 Statistical analysis of frequency maintenance

To determine if DBLα type frequency varied across GhanaMRS surveys, we performed (for each DBLα type) a chi-squared test of association between the presence/absence of that type and the survey. We used a Bonferroni correction to control the family-wise error rate at 0.05; DBLα types that had a p-value below this threshold exhibited significant variation between surveys.

To determine if there was any evidence for balancing selection in the frequency profiles of a single isolate, we fit a binomial regression to the counts of each rarity (low, medium, high, extreme) against the total number of DBLα types in each isolate, separated by ups group and surveys. Significant underdispersion would then indicate the presence of a balancing selection force maintaining the proportions of each frequency at fixed levels.

### 4.9 Determination of DBLα-*var* relationships

For two locations (Ghana and Malawi), *var* gene sequences were available from assemblies generated by [21]. DBLα-*var* relationships were determined using complete *var* exon 1 sequences that are bounded by an N-terminal segment (NTS) and a transmembrane region (TM) on the 5’ and 3’ ends of exon 1, respectively [26]. Briefly, using *vsearch* [74], DBLα types were globally aligned to *var* exon 1 sequences from the same location (e.g., Malawi DBLα types to Malawi *var* exon 1). Given that DBLα types were generated from clustering at a 96% nucleotide identity threshold, these DBLα types were aligned to *var* exon 1 sequences, retaining alignments that meet the same threshold of 96% identity, calculated over the alignment length and excluding terminal gaps *(--iddef 2*). The relationship between a DBLα type and distinct *var* exon 1 was determined based on the number of unique *var* exon 1 sequences sharing a same DBLα type (e.g., a 1-to-*n* DBLα-*var* relationship is defined as a DBLα type found in *n* unique *var* exon 1).

For each group of *var* exon 1 that share a same DBLα type, an all *vs* all sequence alignment of *var* exon 1 sequences in the group was performed using the *allpairs_global* option within *vsearch* [74] and set to include all pairwise alignments *(--acceptall*). Pairwise nucleotide identities were estimated based on calculations over whole alignment lengths, including terminal gaps (*--iddef 1*), to account for differences in pairs of *var* exon 1 of variable lengths.

### 4.10 Search for sequence homology to other DBLα types or *var* genes

#### 4.10.1 *Var* genes in association with selective sweeps on specific chromosomes

Published work reported conserved *var* genes on chromosomes 4, 6, 7, and 8 associated with selective sweep events, potentially due to antimalarial drug resistance or other factors. Accession numbers of these genes were obtained from the author [5,21] and used as reference. Using *blastn* [73], DBLα types were searched against these reference sequences and hits from alignments were reported (≥96% nucleotide identity, ≥95% query coverage).

#### 4.10.2 *Var* genes in primate *Plasmodium* species

*Var* genes from three *Plasmodium* species, *P*. *praefalciparum*, *P*. *reichenowi*, and *P*. *gaboni*, were downloaded from PlasmoDB [2] and used as reference. Using *blastn* [73], DBLα types were searched against these reference sequences and hits from alignments were reported (≥96% nucleotide identity, ≥95% query coverage).

#### 4.10.3 Globally-conserved DBLα types or *var* genes

The 100 most frequent DBLα sequences reported in the global analysis by Tonkin-Hill et al. [28] was used as reference. Using *blastn* [73], DBLα types were searched against these reference sequences and hits from alignments were reported (≥96% nucleotide identity, ≥95% query coverage). The same search parameters and thresholds were applied in searching for homologs to *var* gene sequences of PFGA01_060022400 and PF3D7_0617400, representing conserved *var* genes that are almost identical in sequence as reported by Dimonte et al. [29], as well as to *var* gene sequence PF3D7_0809100, shown by Zanghì et al. [46] to be expressed at the sporozoite stage.

## Supporting information

S1 Text Study of DBLα types and frequencies in Bongo, Ghana. (PDF)

S2 Text Description of the *cUps* algorithm for classifying malaria *var* genes into ups groups. (PDF)

S3 Text Study of DBLα types and frequencies in Africa. (PDF)
