# Supplementary material for: A paradoxical population structure of var DBLα types in Africa
Source: PLoS Pathog. 2025 Feb 4;21(2):e1012813. doi: 10.1371/journal.ppat.1012813 (PMC11793742; doi:10.1371/journal.ppat.1012813)
Supplement: S1 Text — (PDF) [file ppat.1012813.s001.pdf]

S1 Text. Study of DBLα types and frequencies in Bongo, Ghana

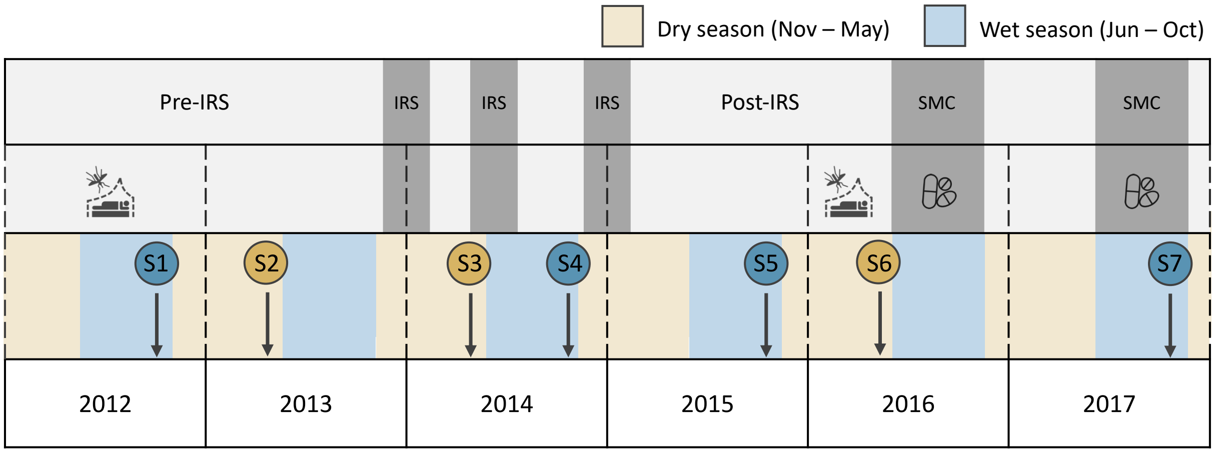

Fig A. Schedule of seven age-stratified cross-sectional surveys in the Malaria Reservoir Study (MRS) conducted in Bongo, Ghana. The study area is typically characterised by high seasonal malaria transmission (prevalence ≥35% at baseline, i.e. S1). Wet- (October) and dry- (May/June) season surveys (S1 to S7) are represented by the blue and yellow circles, respectively. Interventions (i.e., long-lasting insecticidal nets (LLINs), indoor residual spraying (IRS), and seasonal malaria chemoprevention (SMC)) are indicated. Figure was adapted from (1,2). The "Mosquito Net" icon by Luis Prado, from the nounproject.com CC BY 3.0. (https://thenounproject.com/icon/mosquito-net-811132/).

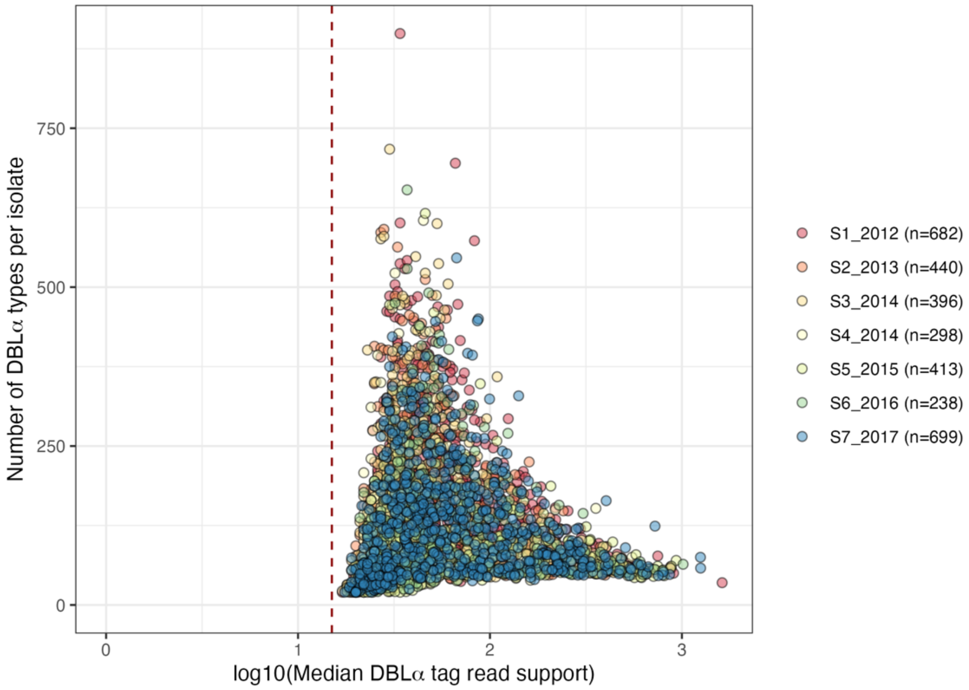

Fig B. Relationship between sequencing depth and number of DBLα types per isolate. For isolates in the GhanaMRS dataset, we calculated the median read support per isolate. The scatter plot shows that higher sequencing depth did not lead to a greater number of types per isolate. The red, dashed line indicates the minimum support threshold (15 reads) applied by the DBLaCleaner pipeline.

Table A. Details on participants, microscopic characteristics, and DBLα types in the Malaria Reservoir Study (MRS) surveys.

Parasitological parameters	Survey 1 (S1)	Survey 2 (S2)	Survey 3 (S3)	Survey 4 (S4)	Survey 5 (S5)	Survey 6 (S6)	Survey 7 (S7)
	Oct (2012)	May/Jun (2013)	May/Jun (2014)	Oct (2014)	Oct (2015)	May/Jun (2016)	Oct (2017)
**Number of participants[a]**	1,923	1,902	1,822	1,866	2,022	2,091	1,915
**Isolates with microscopically positive (prevalence[b])**	808 (42.0)	513 (27.5)	535 (29.4)	430 (23.0)	545 (27.0)	272 (13.0)	789 (41.2)
**Isolates with > 0 DBLα data[c]**	742 (91.8)	468 (91.2)	488 (91.2)	386 (89.8)	508 (93.2)	261 (96.0)	759 (96.2)
**Isolates with ≥ 20 DBLα data[c]**	682 (84.4)	440 (85.8)	396 (74.0)	298 (69.3)	413 (75.8)	238 (87.5)	699 (88.6)
**Total DBLα types**				62,158			
**upsA**				3,370 (5.4)			
**upsB**				35,207 (56.6)			
**upsC**				23,581 (37.9)			
**Number of DBLα types per survey**	33,739	26,628	24,057	15,749	18,690	17,259	28,164
**upsA**	2,400 (7.1)	2,017 (7.6)	1,960 (8.1)	1,605 (10.2)	1,794 (9.6)	1,629 (9.4)	2,252 (8.0)
**upsB**	19,347 (57.3)	15,270 (57.3)	13,730 (57.1)	9,133 (58.0)	10,417 (55.7)	9,635 (55.8)	15,919 (56.5)
**upsC**	11,992 (35.5)	9,341 (35.1)	8,367 (34.8)	5,011 (31.8)	6,479 (34.7)	5,995 (34.7)	9,993 (35.5)
**Number of DBLα types per isolate**							
**Mean**	165.7	161.9	155.2	107.4	98.3	146.5	126.2
**Median**	139.0	138.0	115.0	76.0	75.0	112.5	103.0
**Min**	20	20	21	20	20	21	20
**Max**	899	591	717	605	616	653	546

[a] Number of participants surveyed that were analysed by microscopy.

[b] Data reflect the number (% (n/N)) of participants sampled that were microscopically positive for an asymptomatic *P. falciparum* infection (including mixed *P. falciparum* infections) relative to the number of participants surveyed in the total population or by the age groups presented.

[c] Data reflect the number (% (n/N)) of microscopic *P. falciparum* isolates that had DBLα sequence data relative the number of participants surveyed that were microscopically positive for *P. falciparum* (including mixed *P. falciparum* infections) in the total population. Isolates that had low DNA and/or sequencing quality (i.e., < 20 DBLα types) were removed from downstream analyses (see Methods for additional details).

Table B. Classification of DBLα types in the Malaria Reservoir Study (MRS) surveys into ups groups and frequency classes. Survey-specific frequencies were binned into frequency classes with ranges given in interval notations: *low* (0%, 1%), *moderate* [1%, 5%), *high* [5%, 10%), and *extreme* [10%, 100%].

**Malaria Reservoir Study (MRS) surveys**	**S1**	**S2**	**S3**	**S4**	**S5**	**S6**	**S7**
	**Oct (2012)**	**May/Jun (2013)**	**May/Jun (2014)**	**Oct (2014)**	**Oct (2015)**	**May/Jun (2016)**	**Oct (2017)**
**Number of DBLα types per survey** [a]	33,739	26,628	24,057	15,749	18,690	17,259	28,164
upsA[b]	2,400 (7.1)	2,017 (7.6)	1,960 (8.1)	1,605 (10.2)	1,794 (9.6)	1,629 (9.4)	2,252 (8.0)
(0%, 1%)[c]	1,599 (66.6)	1,287 (63.8)	1,169 (59.6)	939 (58.5)	1,268 (70.7)	916 (56.2)	1,542 (68.5)
[1%, 5%)[c]	664 (27.7)	587 (29.1)	650 (33.2)	580 (36.1)	463 (25.8)	574 (35.2)	620 (27.5)
[5%, 10%)[c]	100 (4.2)	105 (5.2)	106 (5.4)	71 (4.4)	51 (2.8)	103 (6.3)	72 (3.2)
[10%, 100%)[c]	37 (1.5)	38 (1.9)	35 (1.8)	15 (0.9)	12 (0.7)	36 (2.2)	18 (0.8)
upsB[b]	19,347 (57.3)	15,270 (57.3)	13,730 (57.1)	9,133 (58.0)	10,417 (55.7)	9,635 (55.8)	15,919 (56.5)
(0%, 1%)[c]	17,668 (91.3)	13,751 (90.1)	11,812 (86.0)	7,797 (85.4)	9,877 (94.8)	8,142 (84.5)	14,795 (92.9)
[1%, 5%)[c]	1,624 (8.4)	1,458 (9.5)	1,865 (13.6)	1,306 (14.3)	514 (4.9)	1,441 (15.0)	1088 (6.8)
[5%, 10%)[c]	36 (0.2)	43 (0.3)	35 (0.3)	18 (0.2)	17 (0.2)	38 (0.4)	21 (0.1)
[10%, 100%)[c]	19 (0.1)	18 (0.1)	18 (0.1)	12 (0.1)	9 (0.1)	14 (0.1)	15 (0.1)
upsC[b]	11,992 (35.5)	9,341 (35.1)	8,367 (34.8)	5,011 (31.8)	6,479 (34.7)	5,995 (34.7)	9,993 (35.5)
(0%, 1%)[c]	11,029 (92.0)	8,460 (90.6)	7,246 (86.6)	4,321 (86.2)	6,114 (94.4)	5,141 (85.8)	9,269 (92.8)
[1%, 5%)[c]	918 (7.7)	832 (8.9)	1,075 (12.8)	666 (13.3)	340 (5.2)	814 (13.6)	690 (6.9)
[5%, 10%)[c]	28 (0.2)	30 (0.3)	29 (0.3)	14 (0.3)	13 (0.2)	25 (0.4)	21 (0.2)
[10%, 100%)[c]	17 (0.1)	19 (0.2)	17 (0.2)	10 (0.2)	12 (0.2)	15 (0.3)	13 (0.1)

[a] Data reflect DBLα types that have been confirmed through successful categorisation into DBLα domain classes and ups groups.

[b] Data reflects the number (% (n/N)) of DBLα types relative to the number in a survey (i.e., "Number of DBLα types per survey").

[c] Data reflects the number (% (n/N)) of DBLα types relative to the number in a survey and ups group (i.e., "upsA", "upsB", or "upsC").

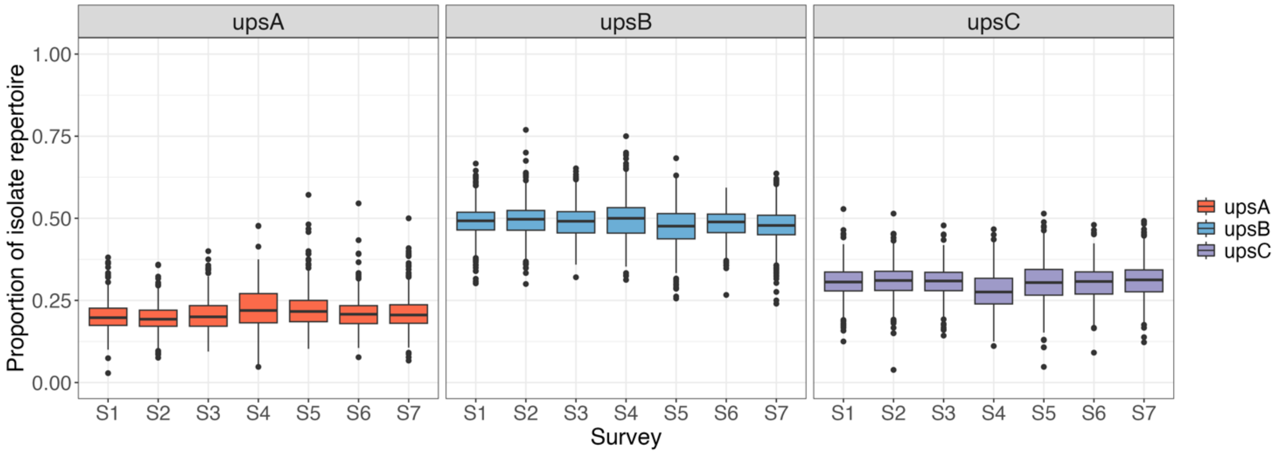

Fig C. Proportions of upsA, upsB, and upsC DBLα types per isolate repertoire [Malaria Reservoir Study (MRS)]. Box plots indicate the medians (centre line), interquartile ranges (IQR, upper and lower quartiles), 1.5× IQR (whiskers), and outliers (points). At the isolate level, the average isolate repertoire consists of 20.9%, 48.6%, and 30.5% of upsA, upsB, and upsC DBLα types, respectively.

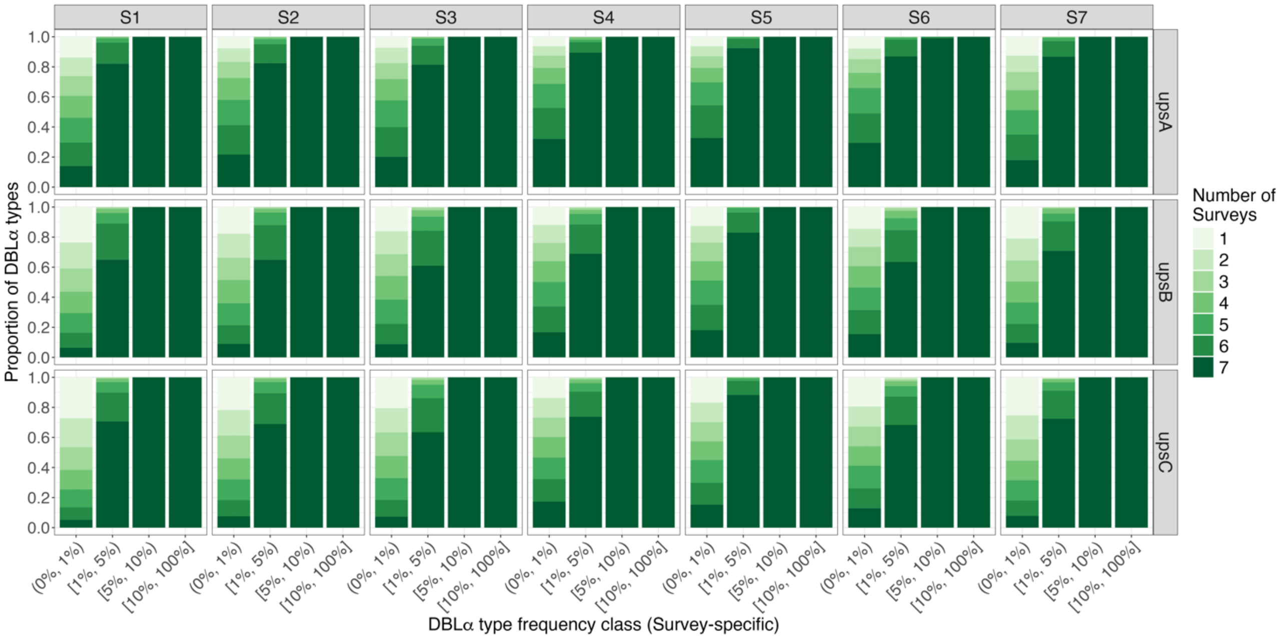

Fig D. Stability of DBLα types through time [Malaria Reservoir Study (MRS)]. Survey-specific frequency classes are shown with ranges given in interval notations of low (0%, 1%), moderate [1%, 5%), high [5%, 10%), and extreme [10%, 100%]. Based on the number of surveys DBLα types are observed in, DBLα types that are present at moderate-to-extreme survey-specific frequencies (≥1%) are shown to also persist through time. This was observed for all three ups groups.

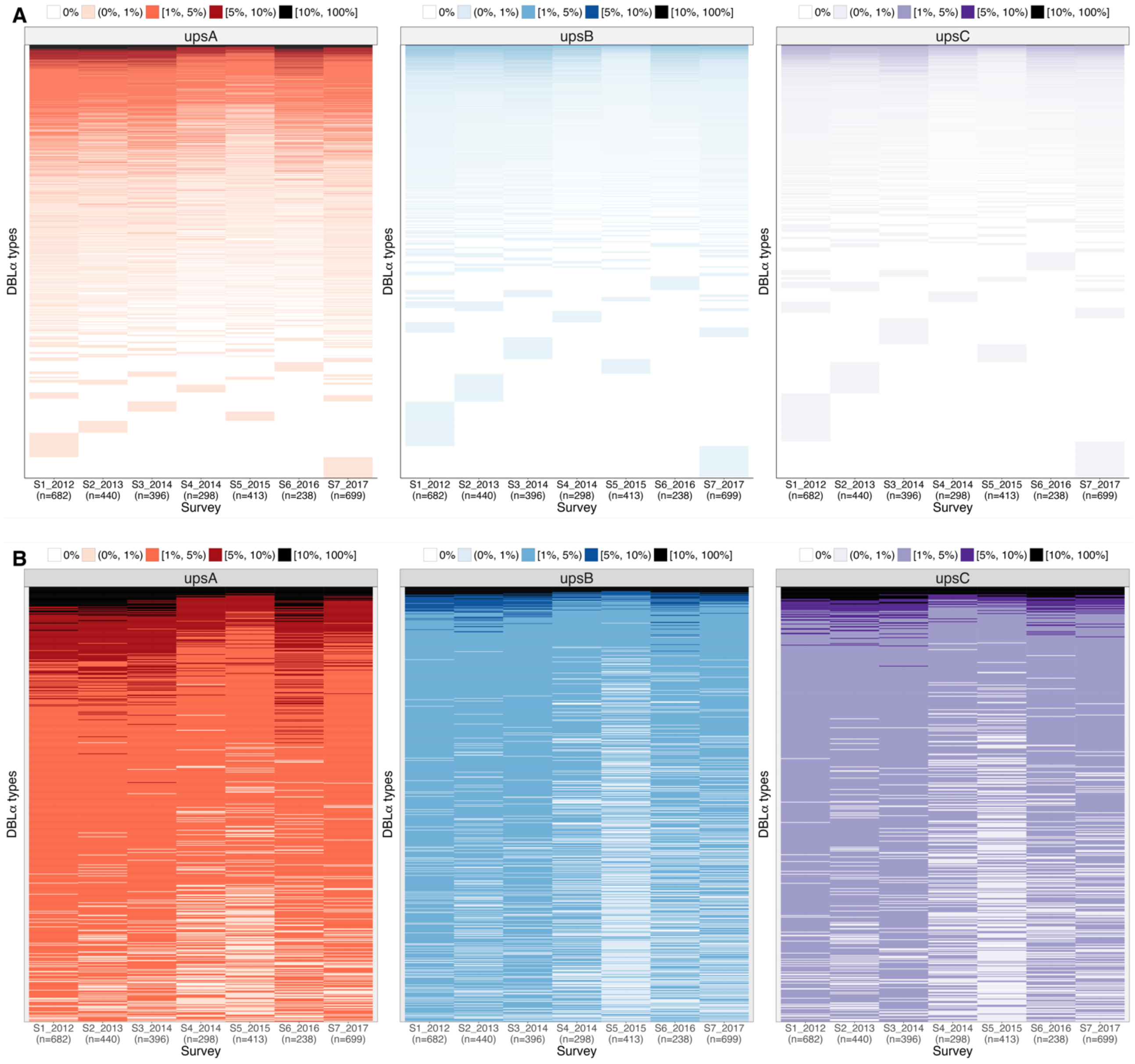

Fig E. Stable DBLα type sequences and frequencies are observed in a local population and through time [Malaria Reservoir Study (MRS)]. Plots show **(A)** all DBLα types and **(B)** DBLα types at ≥1% frequencies (survey-averaged frequencies). Rows represent individual DBLα type in each ups group, ordered in decreasing survey-averaged frequencies. Colours reflect the ups groups and survey-specific frequency classes of DBLα types.

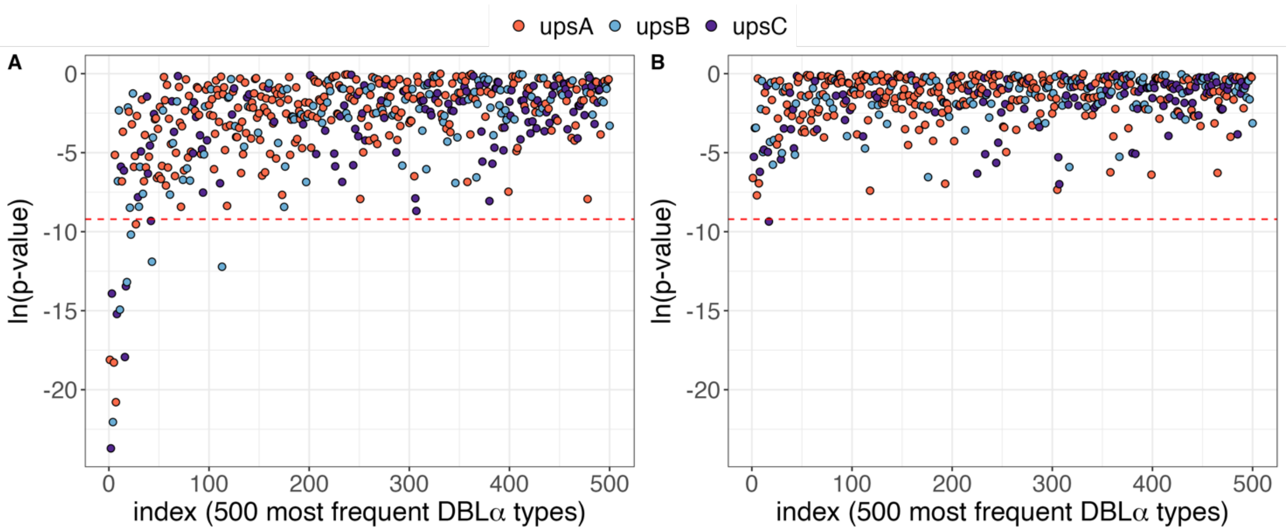

Fig F. (Log) P-values from the chi-squared test of association between presence/absence of each type and survey [Malaria Reservoir Study (MRS)]. Statistically significant variation in the frequencies of the 500 most frequent DBLα types (by survey-averaged frequencies) across surveys were observed, with variations coming exclusively from surveys S4 and S5 that were affected by IRS interventions. Chi-squared tests of association were performed on the 500 most frequent DBLα types in Bongo, Ghana, ordered in decreasing survey-averaged frequencies considering **(A)** seven surveys (S1 to S7), or **(B)** five surveys, excluding IRS-affected surveys S4 and S5. The red horizontal line indicates the Bonferroni correction to control the family-wise error rate at the 0.05 threshold. DBLα types with p-value below this threshold exhibited significant variation between analysed surveys.

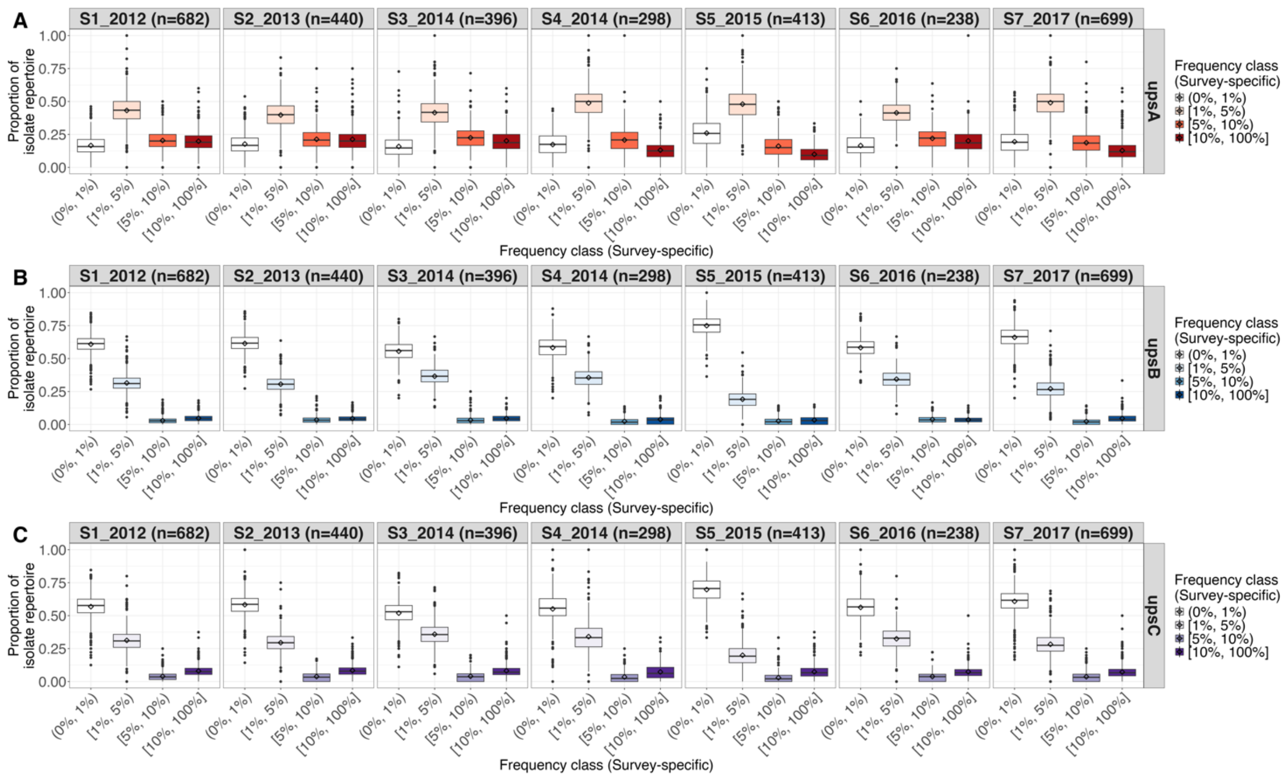

Fig G. Consistent proportions of survey-specific frequency classes within per-isolate frequency profiles [Malaria Reservoir Study (MRS)]. Box plots show distributions of per-isolate proportions for the different survey-specific frequency classes by ups group **(A)** upsA, **(B)** upsB, and **(C)** upsC and by survey (vertical panels), where the 'n' value in the label represents the number of isolates per survey. Box plots indicate median (centre line) and mean (diamond) proportions, interquartile ranges (IQR, upper and lower quartiles), 1.5× IQR (whiskers), and outliers (points). Colours indicate survey-specific frequency classes with ranges given in interval notations of low (0%, 1%), moderate [1%, 5%), high [5%, 10%), and extreme [10%, 100%]. For all ups groups, the per-isolate frequency profiles comprised of a mix of low-to-extreme survey-specific frequency classes and are relatively consistent across isolates within the same survey.

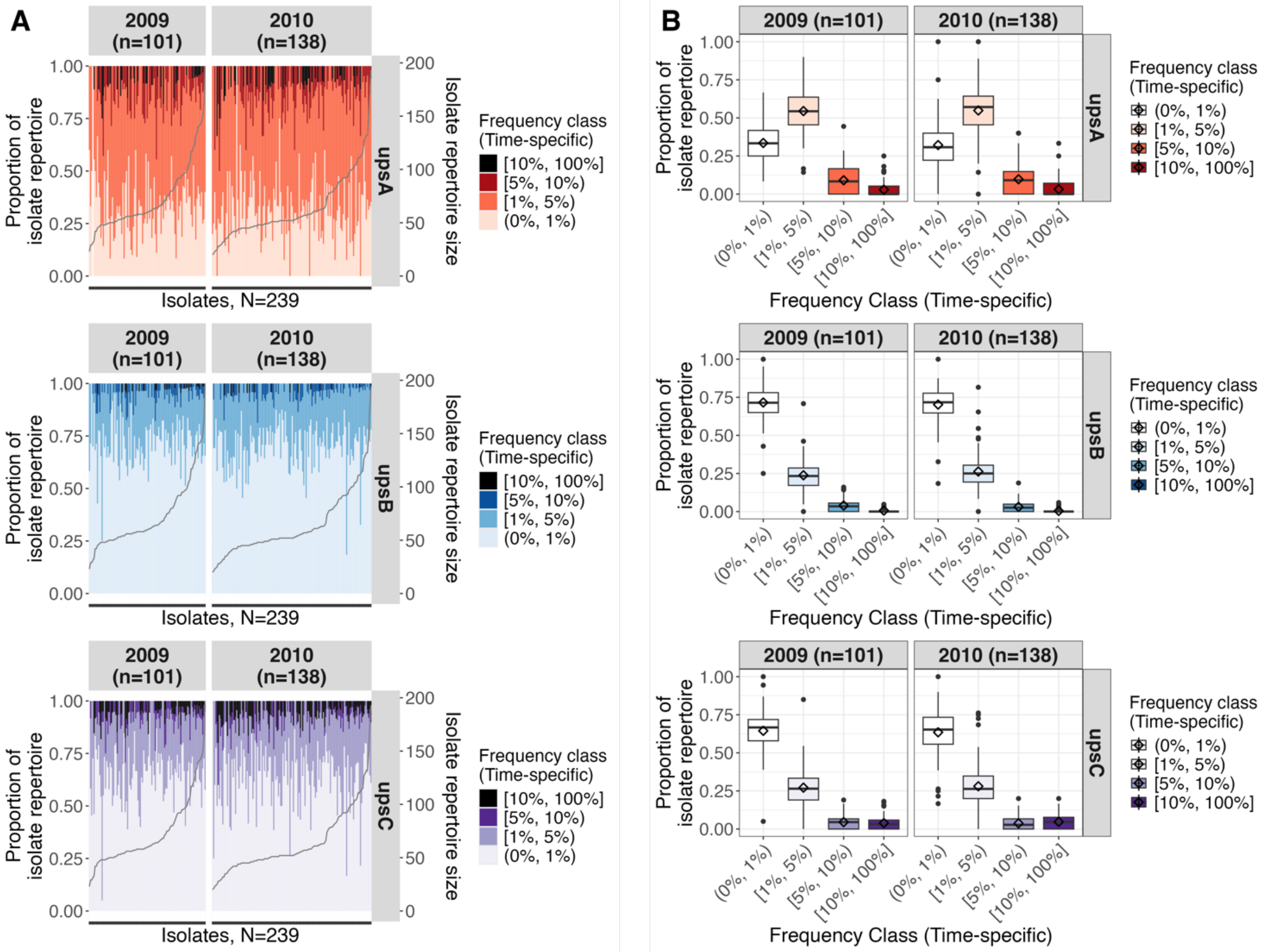

Fig H. Per-isolate frequency profiles show the composition of time-specific frequency classes in every isolate repertoire, using DBLα tag sequences extracted from assembled *var* gene data found in Navrongo, Ghana. For all ups groups (horizontal panels), the per-isolate frequency profiles comprised of a mix of low-to-extreme time-specific frequency classes and are consistent across isolates within the same time point (vertical panels). The 'n' value in labels represents the number of isolates per time point. Colours indicate survey-specific frequency classes with ranges given in interval notations of low (0%, 1%), moderate [1%, 5%), high [5%, 10%), and extreme [10%, 100%]. **(A)** Vertical bars represent individual isolates, ordered in increasing isolate repertoire size (grey line, secondary y-axis). The proportions of time-specific frequency classes within each isolate is shown on the primary y-axis. Isolates on the left side of each panel with smaller isolate repertoire sizes likely represent monoclonal isolates and reflect the composition within actual parasite repertoires. **(B)** Box plots show distributions of per-isolate proportions for the different time-specific frequency classes. Box plots indicate median (centre line) and mean (diamond) proportions, interquartile ranges (IQR, upper and lower quartiles), 1.5× IQR (whiskers), and outliers (points).

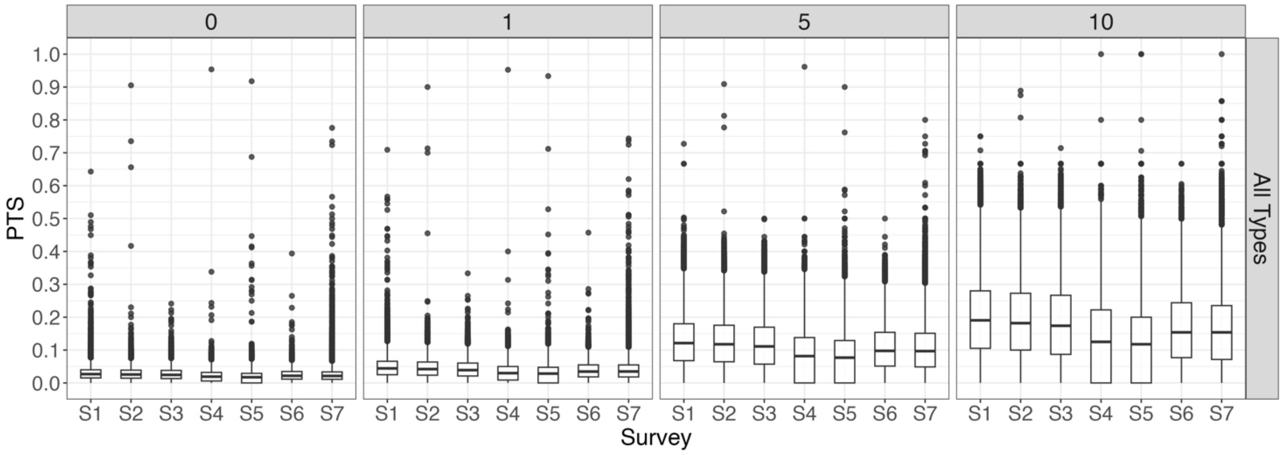

Fig I. Similarity of isolate repertoires by pairwise type sharing (PTS), based on DBLα types present at different minimum survey-specific frequencies [Malaria Reservoir Study (MRS)]. Vertical panels represent the different thresholds of minimum survey-specific frequencies at 0%, 1%, 5%, and 10%. Box plots indicate median values (centre line), interquartile ranges (IQR, upper and lower quartiles), 1.5× IQR (whiskers), and outliers (points). Overall, the distributions of PTS values shift higher as lower-frequency DBLα types are excluded. However, median PTS values remained generally low, indicating a lack of linkage between the DBLα types even at moderate-to-extreme frequencies such that identical sets of types are rarely observed.

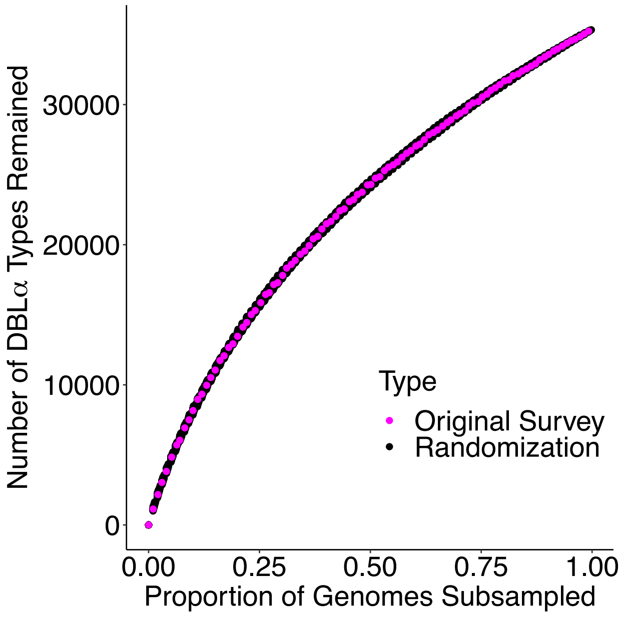

Fig J. Non-linear relationship between the loss of DBLα types and the reduction in the number of parasite genomes. The graph shows that the removal of parasite genomes from the population through intervention does not lead to the loss of the same proportion of DBLα types, as mainly rare DBLα types are initially lost whereas common ones persist until a high proportion of genomes is removed. The survey data used for this subsampling analysis was sampled at the end of wet/high-transmission season in October 2012 from Bongo District in northern Ghana (Survey 1). We used a Bayesian formation of the *var*coding method to calculate the multiplicity of infection of each isolate to further obtain the total number of genomes or parasite census population size as described in (3). The 1,000 randomised realisations of the original survey in sequence were obtained based on the curveball algorithm that keeps isolate lengths and type frequency distribution (4).

REFERENCES

1. Argyropoulos DC, Ruybal-Pesántez S, Deed SL, Oduro AR, Dadzie SK, Appawu MA, et al. The impact of indoor residual spraying on *Plasmodium falciparum* microsatellite variation in an area of high seasonal malaria transmission in Ghana, West Africa. Mol Ecol [Internet]. 2021 Aug 1;30(16):3974–92.

2. Tiedje KE, Oduro AR, Bangre O, Amenga-Etego L, Dadzie SK, Appawu MA, et al. Indoor residual spraying with a non-pyrethroid insecticide reduces the reservoir of *Plasmodium falciparum* in a high-transmission area in northern Ghana. PLOS Global Public Health [Internet]. 2022 May 18;2(5):e0000285.

3. Tiedje KE, Zhan Q, Ruybal-Pésantez S, Tonkin-Hill G, He Q, Tan MH, et al. Measuring changes in *Plasmodium falciparum* census population size in response to sequential malaria control interventions. Elife. 2023 May.

4. Strona G, Nappo D, Boccacci F, Fattorini S, San-Miguel-Ayanz J. A fast and unbiased procedure to randomize ecological binary matrices with fixed row and column totals. Nat Commun. 2014;5(1):4114.
