# Supplementary material for: A paradoxical population structure of var DBLα types in Africa
Source: PLoS Pathog. 2025 Feb 4;21(2):e1012813. doi: 10.1371/journal.ppat.1012813 (PMC11793742; doi:10.1371/journal.ppat.1012813)
Supplement: S3 Text — (PDF) [file ppat.1012813.s003.pdf]

S3 Text. Study of DBLα types and frequencies in Africa

Table A. Dataset sources in Africa and Asia. All datasets were used in the clustering step to generate representative DBLα types. However, further study on DBLα types and frequencies was limited to only African locations with ≥100 isolates. For GhanaMRS isolates, this study was limited to only surveys 1 to 7, excluding the pilot study.

Continent	Location	Year(s) collected	DBLα source^	# Isolates (initial)	# Isolates (≥20 types)	# Sites +	# Years +
Africa	Senegal (West)[1]*	2001-2014	Assembled *var*	91	63		
Africa	Gambia (West)[1]*	2008, 2013-2015	Assembled *var*	64	62		
Africa	Guinea (West)[1]*	2011	Assembled *var*	96	94		
Africa	Mali (West)[1]*	2007, 2012-2014	Assembled *var*	81	75		
Africa	GhanaMRS (West)[2]	2012 (Pilot study)	AmpSeq	161	158		
Africa	GhanaMRS (West)[2]	2012-2017	AmpSeq	3,955	3,166	1	7
Africa	Ghana (West)[1]	2009-2015	Assembled *var*	530	474	2	5
Africa	Nigeria (West)[1]	2012	Assembled *var*	3	3		
Africa	Gabon (West)[3]	2000	AmpSeq	200	176	1	1
Africa	Democratic Republic of Congo (Central)[1]*	2012-2015	Assembled *var*	102	92		
Africa	Malawi (Central)[1]	2011	Assembled *var*	253	246	2	1
Africa	Uganda (East)[4]	2006-2007	AmpSeq	517	499	6	2
Africa	Kenya (East)[1]*	2007, 2009, 2014	Assembled *var*	47	26		
Asia	Bangladesh[1]*	2012	Assembled *var*	49	46		
Asia	Cambodia[1]*	2008-2012	Assembled *var*	624	440		
Asia	Laos[1]*	2011-2012	Assembled *var*	82	79		
Asia	Myanmar[1]*	2011-2013	Assembled *var*	58	56		
Asia	Thailand[1]*	2011-2013	Assembled *var*	142	135		
Asia	Vietnam[1]*	2009-2011	Assembled *var*	128	94		

[1] (1), [2] (2–7), [3] (8), [4] (9)

^ "AmpSeq" refers to amplicon sequencing of the DBLα tag region; "Assembled *var*" refers to DBLα tags extracted from assembled *var* genes.

* Locations were excluded from the study either due to location (Asia is excluded) or due to limitations in the dataset size (i.e., in Africa but # Isolates (final) < 100).

+ Values shown only for datasets retained in final spatial analysis, following the exclusion of individual isolates with < 20 DBLα types and subsequent exclusion of locations with < 100 isolates. "# Sites" and "# Years" refers to specific area and time sampling was performed, based on metadata available by MalariaGen e.g., Pf3k, Pf6 (10).

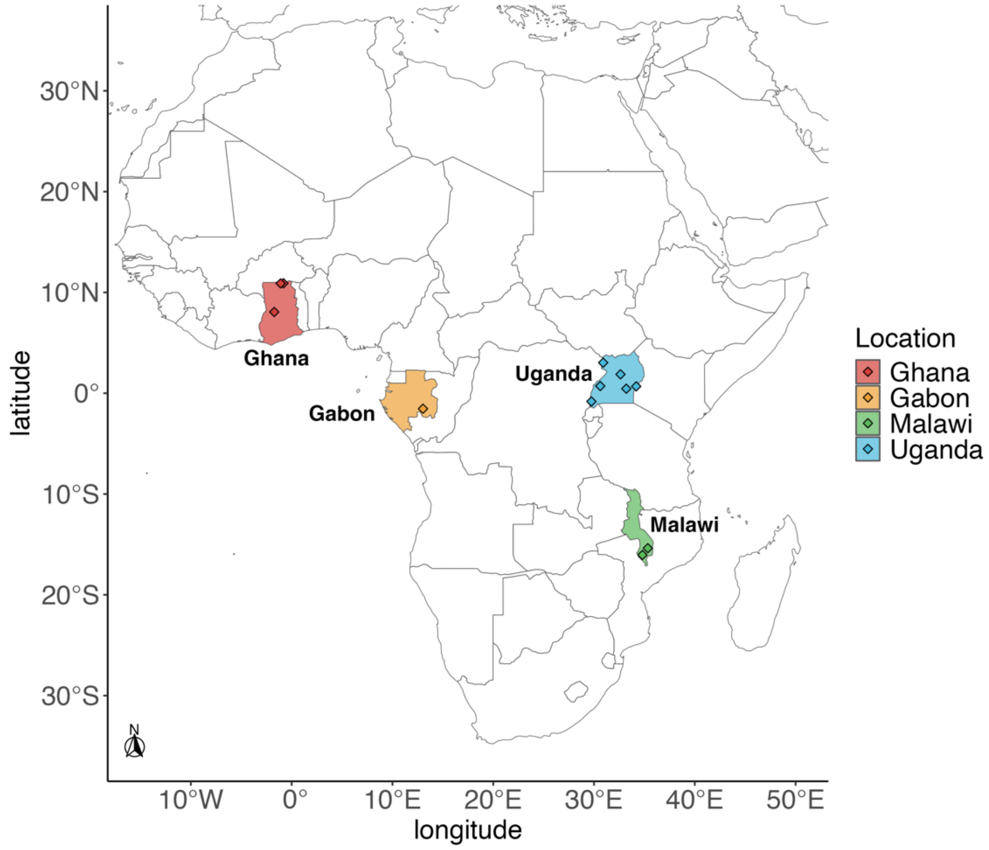

Fig A. Locations (i.e., countries) in Africa included in the geographical analysis of DBLα types and frequencies. Map was made with Natural Earth (http://www.naturalearthdata.com) using the *rnaturalearth* R package (v.0.1.0) (11).

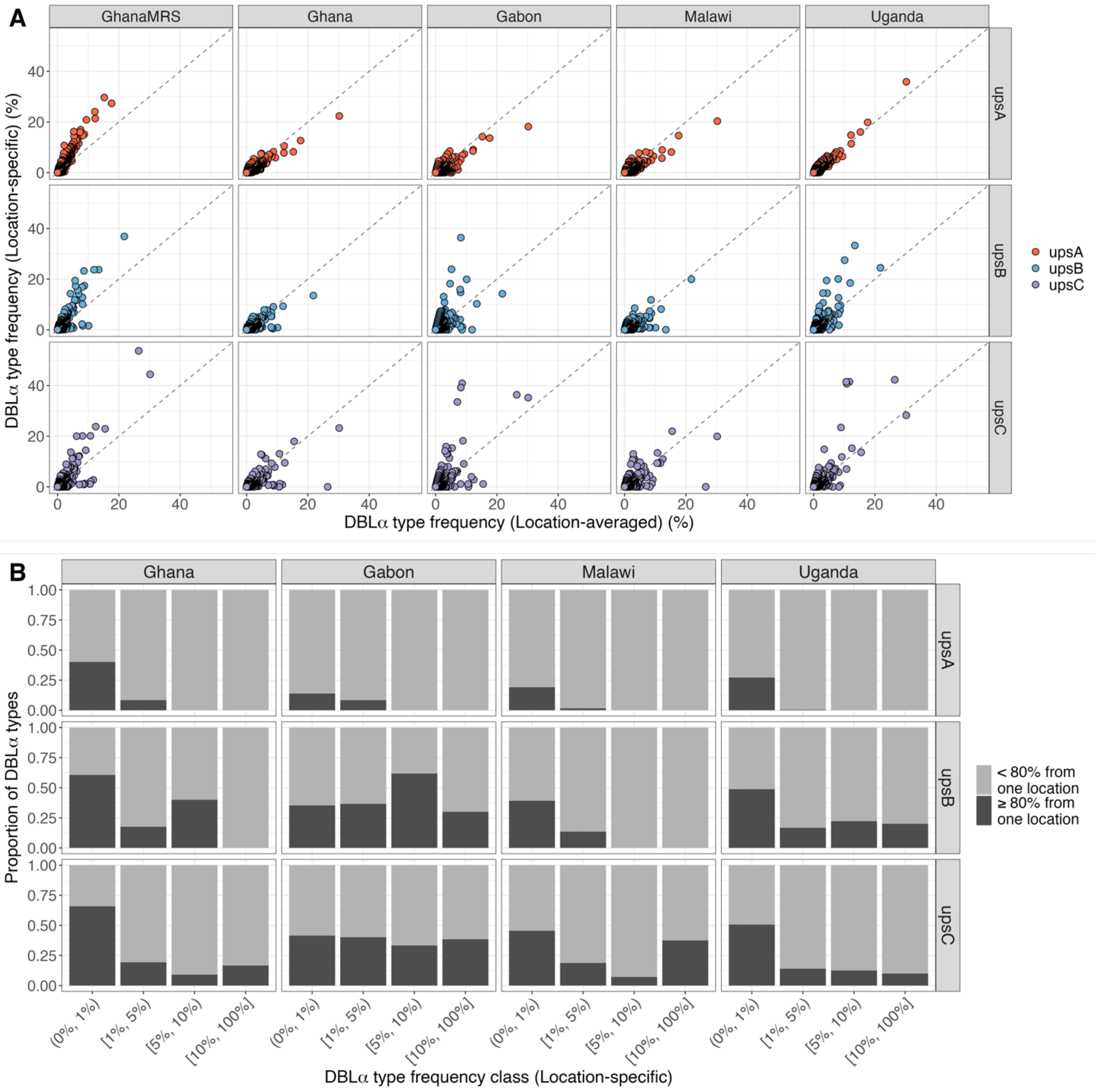

Fig B. Local signatures of DBLα types and frequencies are observed in populations in Ghana, Gabon, Malawi, and Uganda in Africa [geographical analysis]. Local DBLα types are defined as types that are present predominantly in a single location but are absent or found at low frequencies in other locations. These local types appear to be more commonly observed in the upsB and upsC groups. **(A)** Location-specific frequencies (y-axis) are plot against location-averaged frequencies (x-axis) for individual DBLα types (points), coloured by ups groups. Data points strongly deviating from the x=y diagonal suggest that these DBLα types are present at relatively higher frequencies in a local population but may not be across all locations. **(B)** Proportions of DBLα types where a single location is a major source contributor. A location is considered the major source contributor for a DBLα type if ≥80% of the isolates in which the DBLα type is found is from that one same location. Shown here are proportions of DBLα types with a single location as the major source contributor, in every location-specific frequency class with ranges given in interval notations of low (0%, 1%), moderate [1%, 5%), high [5%, 10%), and extreme [10%, 100%]. The "Ghana" location here includes DBLα types present in the combined datasets of "GhanaMRS" and "Ghana".

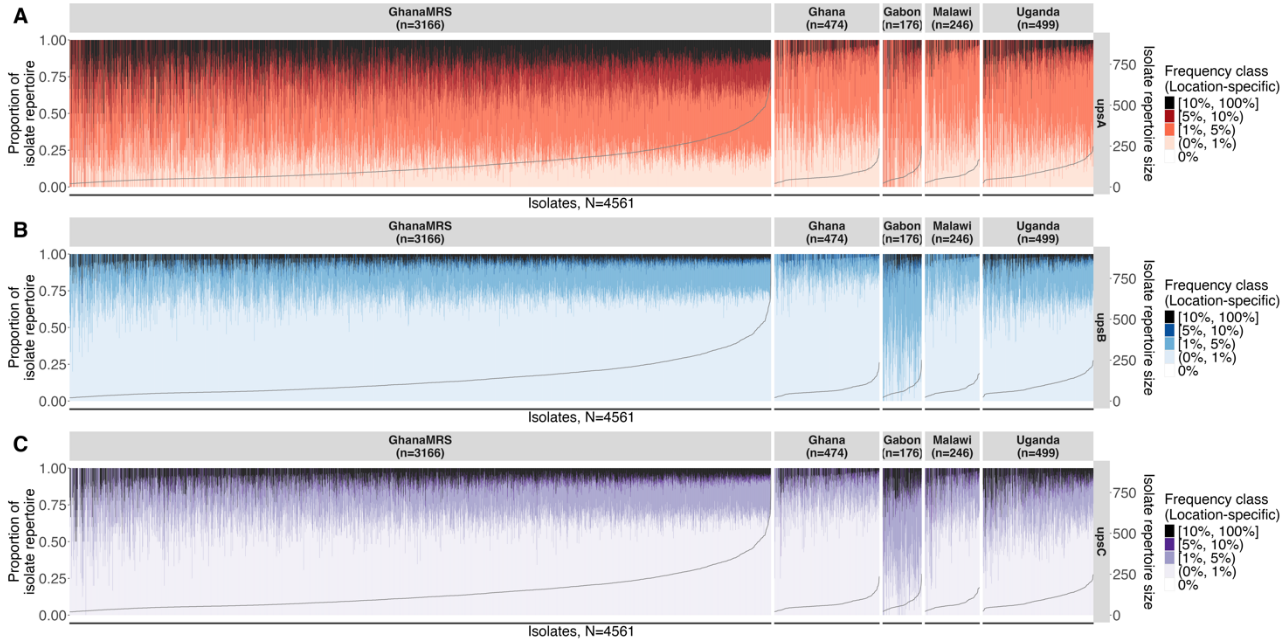

Fig C. Per-isolate frequency profiles for upsA, upsB, and upsC DBLα types within specific locations [geographical analysis]. Similar to that observed for the GhanaMRS dataset, the per-isolate frequency profiles in other African locations for all ups groups also comprised of a mix of low-to-extreme survey-specific frequency classes and are consistent across isolates within the same survey, regardless of isolates' infection complexities. Per-isolate frequency profiles are shown here by ups group **(A)** upsA, **(B)** upsB, and **(C)** upsC and by location (vertical panels), where the 'n' value in the label represents the number of isolates per location. Vertical bars represent individual isolates, ordered in increasing isolate repertoire size (grey line, secondary y-axis). Colours indicate location-specific frequency classes with ranges given in interval notations of low (0%, 1%), moderate [1%, 5%), high [5%, 10%), and extreme [10%, 100%], and the proportions of these frequency classes within each isolate is shown on the primary y-axis. Isolates on the left side of each vertical panel with smaller isolate repertoire sizes likely represent monoclonal isolates and reflect the composition within actual parasite repertoires.

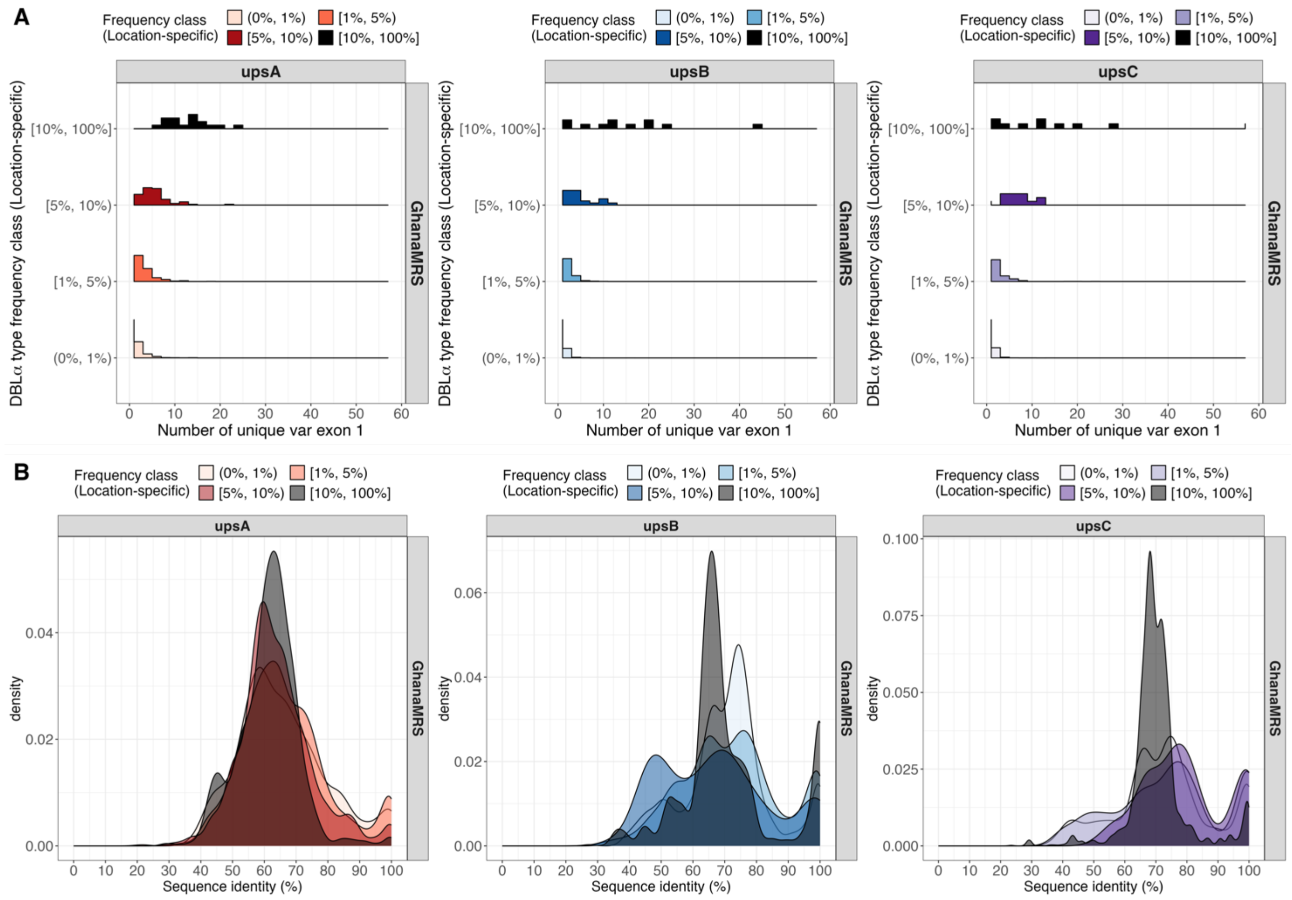

Fig D. Relationship between DBLα types and *var* exon 1 [geographical analysis]. Analysis was performed through sequence alignment of DBLα types found in the GhanaMRS data to *var* exon 1 sequences in Navrongo. Both locations (Bongo and Navrongo) are situated ~30km apart in Ghana. **(A)** Distributions of the number of unique *var* exon 1 sequences (in Navrongo) containing the same DBLα type show that DBLα types at high or extreme frequencies tend to be associated with multiple different *var* exon 1 sequences. **(B)** Distributions of sequence similarity between pairs of aligned *var* exon 1 that share the same DBLα type show peaks at approximately 60% to 80% of pairwise nucleotide identities, suggesting that the majority of these *var* exon 1 represent actual different genes. However, minor peaks on the right side of these distributions, especially for upsB and upsC groups, could suggest that a subset of these *var* exon 1 are alleles of a same *var* gene.

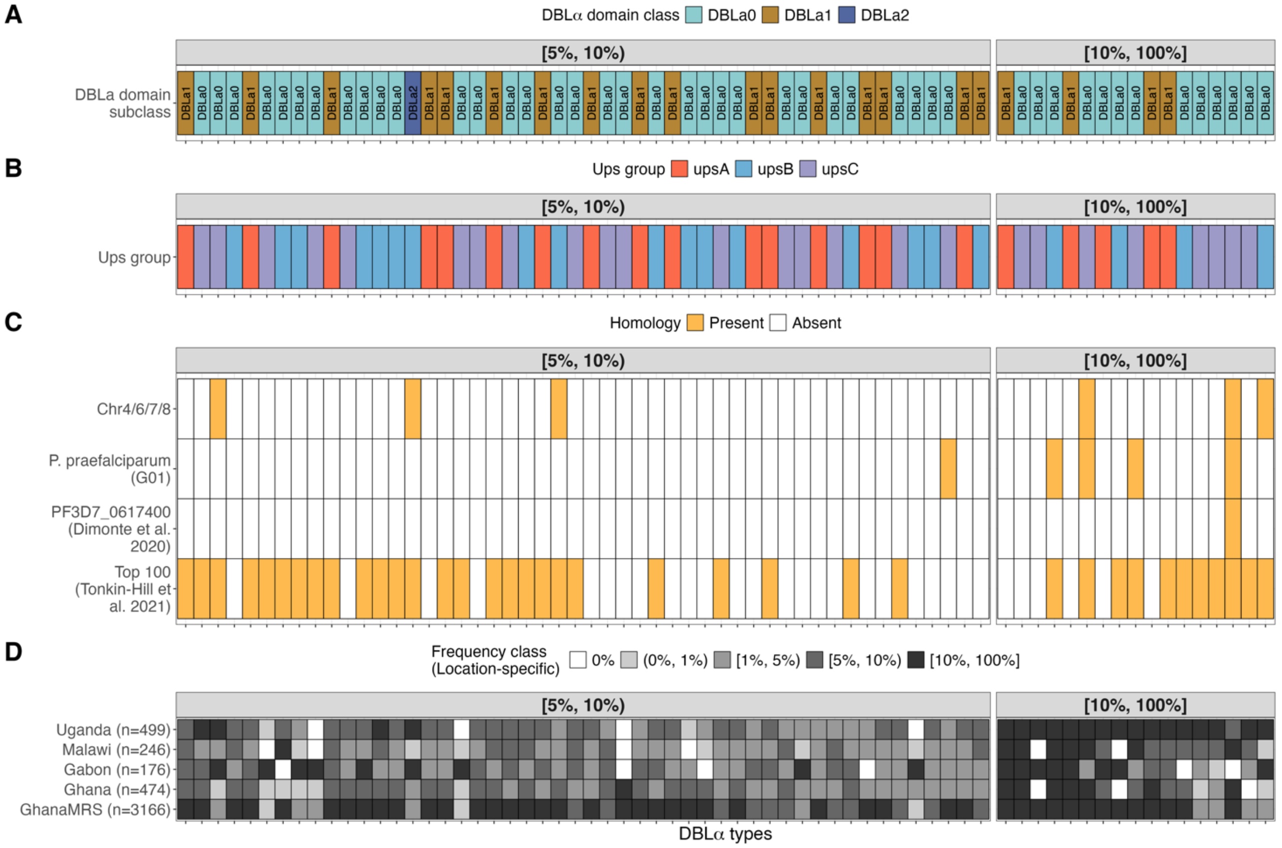

Fig E. Annotation of possible factors maintaining DBLα types with high or extreme frequencies [geographical analysis]. Shown here are DBLα types with high or extreme location-averaged frequency classes (vertical panels), with information on the **(A)** assigned DBLα domain subclass, **(B)** classified ups group, **(C)** sequence homology (presence/absence) to published conserved DBLα tags and *var* genes, and **(D)** location-specific frequency classes, where the 'n' value in the label represents the number of isolates per location. While some factors (e.g., drug resistance selective sweeps, ancestral origins) can explain the reason a few of these DBLα types are maintained in one or many populations, the majority of these higher-frequency DBLα types are still unaccounted for. **Note:** The homolog to the DBLα tag of a conserved *var* gene reported in a Gabonese isolate (12) is present in all locations analysed in this study except in Gabon itself. Located on chromosome 6, this coincides with previous reports of haplotypes in linkage disequilibrium on the same chromosome (13–15), though this *var* gene is located outside of this region's cluster. A possible explanation for the absence of this homolog in the Gabon dataset used in this study may be that the isolates were sampled relatively early in the timeline (year 2000), which precedes the switch to artemisinin (ART)-based combination therapies (ACT) in Africa (16,17), suggesting that the selection for this specific type may not yet have risen in frequency to result in observed fixation in the population at the time.

REFERENCES

1. Otto TD, Assefa SA, Böhme U, Sanders MJ, Kwiatkowski DP, Null N, et al. Evolutionary analysis of the most polymorphic gene family in falciparum malaria [version 1; peer review: 1 approved, 2 approved with reservations]. Wellcome Open Res. 2019;4(193).

2. He Q, Pilosof S, Tiedje KE, Ruybal-Pesántez S, Artzy-Randrup Y, Baskerville EB, et al. Networks of genetic similarity reveal non-neutral processes shape strain structure in *Plasmodium falciparum*. Nat Commun. 2018;9(1):1817.

3. Pilosof S, He Q, Tiedje KE, Ruybal-Pesántez S, Day KP, Pascual M. Competition for hosts modulates vast antigenic diversity to generate persistent strain structure in *Plasmodium falciparum*. PLoS Biol. 2019 Jun 24;17(6):e3000336.

4. Tiedje KE, Oduro AR, Agongo G, Anyorigiya T, Azongo D, Awine T, et al. Seasonal Variation in the Epidemiology of Asymptomatic *Plasmodium falciparum* Infections across Two Catchment Areas in Bongo District, Ghana. The American Society of Tropical Medicine and Hygiene. 2017;97(1):199–212.

5. Ruybal-Pesántez S, Tiedje KE, Pilosof S, Tonkin-Hill G, He Q, Rask TS, et al. Age-specific patterns of DBLα *var* diversity can explain why residents of high malaria transmission areas remain susceptible to *Plasmodium falciparum* blood stage infection throughout life. Int J Parasitol. 2022;

6. Tiedje KE, Oduro AR, Bangre O, Amenga-Etego L, Dadzie SK, Appawu MA, et al. Indoor residual spraying with a non-pyrethroid insecticide reduces the reservoir of *Plasmodium falciparum* in a high-transmission area in northern Ghana. PLOS Global Public Health. 2022 May 18;2(5):e0000285.

7. Tiedje KE, Zhan Q, Ruybal-Pésantez S, Tonkin-Hill G, He Q, Tan MH, et al. Measuring changes in *Plasmodium falciparum* census population size in response to sequential malaria control interventions. Elife [Internet]. 2023.

8. Day KP, Artzy-Randrup Y, Tiedje KE, Rougeron V, Chen DS, Rask TS, et al. Evidence of strain structure in *Plasmodium falciparum var* gene repertoires in children from Gabon, West Africa. Proceedings of the National Academy of Sciences. 2017;114(20):E4103–11.

9. Ruybal-Pesántez S, Tiedje KE, Tonkin-Hill G, Rask TS, Kamya MR, Greenhouse B, et al. Population genomics of virulence genes of *Plasmodium falciparum* in clinical isolates from Uganda. Sci Rep. 2017;7(1):11810.

10. MalariaGen, Ahouidi A, Ali M, Almagro-Garcia J, Amambua-Ngwa A, Amaratunga C, et al. An open dataset of *Plasmodium falciparum* genome variation in 7,000 worldwide samples [version 2; peer review: 2 approved]. Wellcome Open Res. 2021;6:42.

11. South A. Rnaturalearth: world map data from natural earth. R package version 01 0. 2017.

12. Dimonte S, Bruske EI, Enderes C, Otto TD, Turner L, Kremsner P, et al. Identification of a conserved *var* gene in different *Plasmodium falciparum* strains. Malar J. 2020;19(1):194.

13. Amambua-Ngwa A, Park DJ, Volkman SK, Barnes KG, Bei AK, Lukens AK, et al. SNP Genotyping Identifies New Signatures of Selection in a Deep Sample of West African *Plasmodium falciparum* Malaria Parasites. Mol Biol Evol. 2012;29(11):3249–53.

14. Amambua-Ngwa A, Danso B, Worwui A, Ceesay S, Davies N, Jeffries D, et al. Exceptionally long-range haplotypes in *Plasmodium falciparum* chromosome 6 maintained in an endemic African population. Malar J. 2016;15(1):515.

15. Amambua-Ngwa A, Button-Simons KA, Li X, Kumar S, Brenneman KV, Ferrari M, et al. Chloroquine resistance evolution in *Plasmodium falciparum* is mediated by the putative amino acid transporter AAT1. Nat Microbiol. 2023;8(7):1213–26.

16. Eastman RT, Fidock DA. Artemisinin-based combination therapies: a vital tool in efforts to eliminate malaria. Nat Rev Microbiol. 2009;7(12):864–74.

17. Bosman A, Mendis. KN. A Major Transition in Malaria Treatment: The Adoption and Deployment of Artemisinin-Based Combination Therapies. In: Breman JG, Alilio MS, White NJ, editors. Defining and Defeating the Intolerable Burden of Malaria III: Progress and Perspectives: Supplement to Volume 77(6) of American Journal of Tropical Medicine and Hygiene. Northbrook (IL): American Society of Tropical Medicine and Hygiene; 2007.
